# Factors relevant to atrial ^18^F-fluorodeoxyglucose uptake in atrial fibrillation

**DOI:** 10.1007/s12350-018-1387-4

**Published:** 2018-08-07

**Authors:** Boqia Xie, Bi-Xi Chen, Jiao-Yan Wu, Xingpeng Liu, Min-Fu Yang

**Affiliations:** 1 Cardiac Center, Beijing Chaoyang Hospital, Capital Medical University, Beijing, China; 2 Department of Nuclear Medicine, Beijing Chaoyang Hospital, Capital Medical University, 8th Gongtinanlu Rd, Chaoyang District, Beijing, 100020 China

**Keywords:** Atrial fibrillation, fluorodeoxyglucose, positron emission tomography/computed tomography

## Abstract

**Background:**

This retrospective study was designed to explore the factors relevant to increased atrial ^18^F-fluorodeoxyglucose (FDG) uptake in patients with atrial fibrillation (AF) who had undergone routine whole-body positron emission tomography/computed tomography (PET/CT) imaging.

**Methods and Results:**

Forty-eight consecutive AF patients (32 persistent, 16 paroxysmal) were identified from our routine FDG PET/CT database. Twenty-two control subjects were selected to establish the normal range of FDG uptake (maximum standardized uptake value, SUV_max_) in target tissues. A target-to-background ratio (TBR) was calculated to determine abnormal uptake in the atrium and atrial appendage (AA). Univariate comparisons and multivariate regression analyses were conducted to explore the factors associated with the increased FDG accumulation in the atrium and AA. Seventeen AF patients, all with persistent AF, had increased atrial FDG uptake. Most of them (14, or 82.4%) had increased uptake in the right atrium. Eleven AF patients, 9 with persistent AF, had increased uptake in the AA, and bilateral AAs were equally involved. Multivariate logistic regression analyses identified that female gender, persistent AF, and activity in epicardial adipose tissue (EAT) were independent factors predicting the increased activity of the atrium; also, SUV_max_ of the left ventricle was found for the AA. In addition, multivariate linear regression analyses showed that EAT activity was the only independent variable linearly correlated with the activity of the atrium and AA.

**Conclusions:**

Atrial uptake was present in persistent AF and localized mainly in the right atrium, whereas bilateral AAs could be equally involved. Multiple factors contributed to the increased activity in atrium; in particular, the EAT activity was independently correlated with the activity of the atrium and AA.

**Electronic supplementary material:**

The online version of this article (10.1007/s12350-018-1387-4) contains supplementary material, which is available to authorized users.

## Introduction

Some case reports and a retrospective study of atrial fibrillation (AF) have found increased uptake of ^18^F-fluorodeoxyglucose (FDG) in the atria of patients who had undergone whole-body positron emission tomography (PET).^1^–^5^ Under fasting conditions, in which the routine FDG imaging was conducted, physiologic uptake in the myocardium was significantly suppressed and atrial FDG uptake was rarely seen in normal subjects. Hence, atrial FDG uptake in AF patients is likely attributable to pathologic mechanisms. Identification of the factors relevant to the abnormal uptake may be beneficial for elucidating the pathogenesis of AF.

Recent advances have revealed that inflammation is related to the initiation and persistence of AF.^6^,^7^ On the one hand, inflammation can initiate AF. Inflammatory cells or mediators have been observed in cardiac tissue, epicardial adipose tissue (EAT), hematopoietic tissue, and the systemic circulation.^7^–^10^ Inflammatory cells and mediators may induce changes of atrial electrophysiology and structure and eventually the occurrence of AF. On the other hand, AF can generate an inflammatory response, and elevated inflammatory markers have been detected during the persistence of AF. Moreover, intensive studies have shown that activated inflammatory cells exhibit enhanced glucose uptake, which can be detected by FDG imaging.^11^ Accordingly, it is reasonable to speculate that the increased atrial uptake of FDG in AF is representative of or associated with inflammation.

In addition to inflammatory cells, cardiomyocytes may take up more glucose in conditions of ischemia and overloading, which have been shown to be present in AF. Moreover, some other pathologic conditions (diabetes, malignancy, and so on) and demographic parameters may be associated with the atrial uptake as well. Therefore, the aim of this study was to analyze the factors relevant to abnormal atrial FDG uptake in AF subjects. In particular, we intended to investigate the relationship between atrial FDG accumulation and systemic and local inflammation as evaluated by FDG PET/CT.

## Methods

### Study Patients

This retrospective study was approved by the Institutional Ethics Committee of Beijing Chaoyang Hospital. Informed consents were waived owing to the study’s retrospective nature. In searching our whole-body FDG PET/CT database (4415 patients) between June 2010 and October 2017, consecutive patients with a diagnosis of AF prior to FDG imaging were identified. The demographic and clinical information were obtained by reviewing the medical records. Sixty AF patients were initially identified, but 12 were excluded for the following reasons: (1) an echocardiogram or other medical information was not available (9 cases); (2) the patient had a history of splenectomy (1 case); and (3) there was suspicion of a metastatic tumor in the spleen (1 case) or lumbar vertebrae (1 case). The diagnosis of persistent AF was confirmed by 24-hour Holter monitoring or multiple electrocardiographic (ECG) records; 34 of them had a history of AF, and the remaining 2 were newly diagnosed. As for patients with paroxysmal AF, all except one had ECG records showing paroxysmal AF during hospitalization. The interval between the latest ECG record and PET/CT imaging was 6 (mean 2-9) days. None of the remaining 48 patients had undergone radio- and/or chemo-therapy in the 6 months prior to PET/CT imaging. Furthermore, a group of control subjects (22 individuals) was selected from the same database to establish the normal range of FDG uptake in the atrium and other target organs/tissues. The inclusion criteria of the control group were as follows: (1) matched to AF patients for age, gender, body mass index (BMI), and diabetes mellitus; (2) no history of AF; (3) no history of cardiovascular disease (coronary artery disease, congenital heart disease, hypertension, pulmonary hypertension, and so on); (4) no history of malignancy; and (5) no malignant or inflammatory findings on PET/CT imaging.

### Echocardiography

The echocardiographic results of all patients within 2 weeks of PET/CT imaging were retrospectively reviewed. Left atrial volume index (LAVI) and right atrial (RA) area were calculated based on concurrent biplane two-dimensional echocardiography (Simpson’s method).^12^,^13^

### PET/CT Imaging

PET/CT scans were performed on a GE Discovery STE device using a standard protocol. Patients fasted for 13 to 17 hours (mean 15.0 ± 0.9 hours) and had a blood glucose level of 111.6 ± 32.4 mg/dL before FDG administration. Whole-body PET/CT scans were obtained 53 to 83 minutes (mean 68.2 ± 8.3 minutes) after intravenous injection of 3.7 MBq/kg of FDG. CT parameters were as follows: 140 kV, 120 mA, pitch 1.375, 16 × 0.625 mm collimation, and section width 5 mm. PET parameters were as follows: 2.5 min/bed from the skull base to the upper thighs and 5 min/bed for head in three-dimensional (3D) mode. Attenuation-corrected PET images (voxel size, 3.9 × 3.9 × 3.3 mm) were reconstructed from the CT data using a 3D ordered-subset expectation maximization algorithm (14 subsets, 2 iterations). Integrated PET and CT images were obtained automatically on AW VolumeShare2 (GE Healthcare).

**Figure 1:**
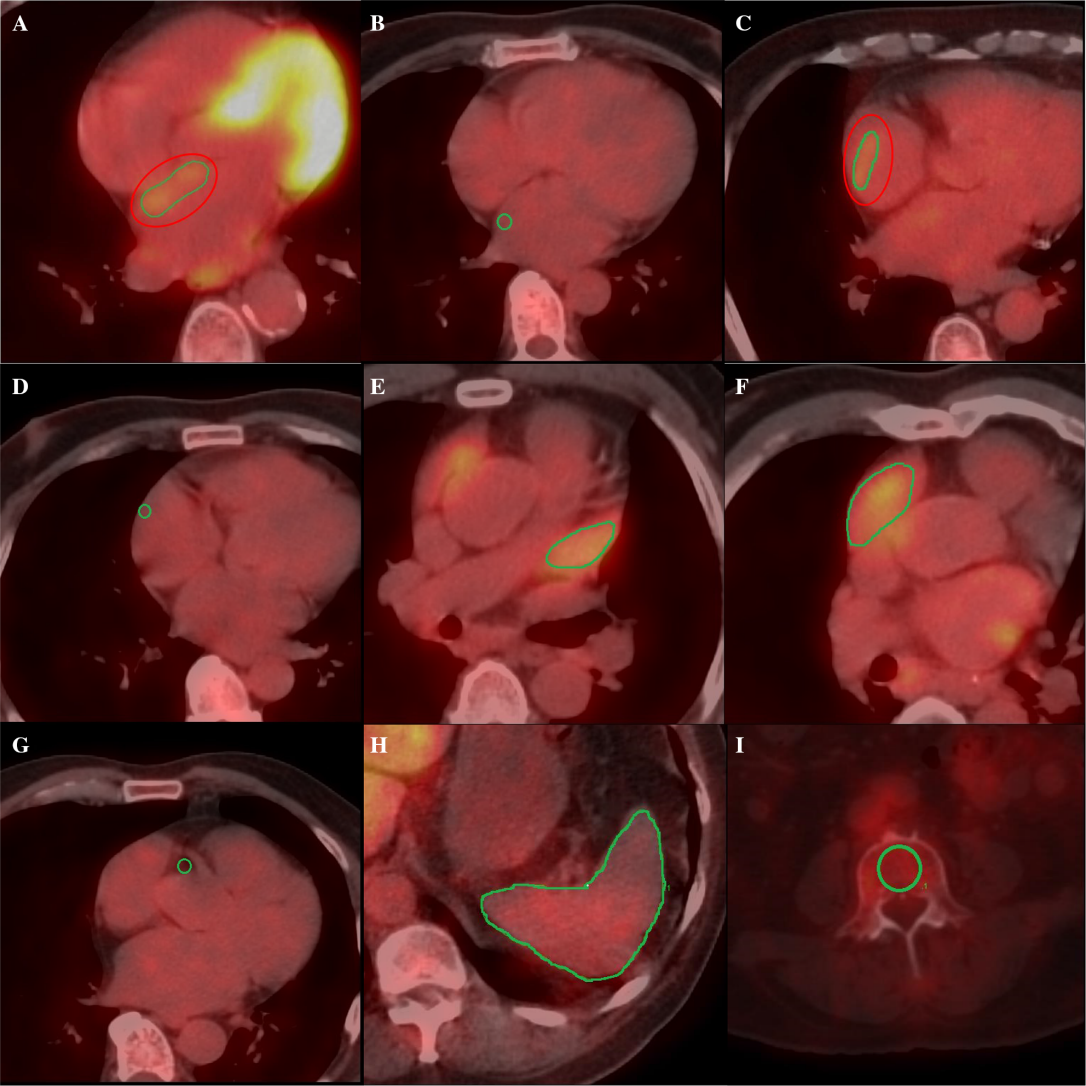
Representative axial positron emission tomography/computed tomography (PET/CT) images showing examples of regions of interest (ROIs) in the left atrial wall with (**A**) and without (**B**) visual uptake, right atrial wall with (**C**) and without (**D**) visual uptake, left atrial appendage (**E**), right atrial appendage (**F**), right coronary artery (**G**), spleen (**H**), and bone marrow (**I**)

Two nuclear physicians (BXC and JYW) discussed the details for evaluating the PET/CT images together and then measured the images separately. All of the datasets were processed twice by one observer (BXC) and once by another observer (JYW) for the evaluation of intra- and inter-observer reproducibility. Both observers were blinded to all other clinical information. FDG uptake in the atrial wall, atrial appendage (AA), hematopoietic tissues (spleen and bone marrow), and EATs were measured, respectively (Figure 1).

Considering the activity of atria and AAs may have varying pathologic significance, they were analyzed separately. All of the PET measurements were guided by CT. For atrial measurement, on each PET and CT registered transaxial image, a region of interest (ROI) was carefully placed on the atrial wall showing visible uptake (higher than the activity of the blood pool). The maximum standardized uptake value (SUV_max_) out of all slices was selected to represent the activity of the atrium. If no visible uptake could be appreciated, a circular ROI 5 mm in diameter was placed on the right lateral wall of the LA at the level of the right inferior pulmonary vein and on the right lateral wall of the RA at the level of aortic root. The activity of the AA was obtained by placing a ROI around the AA on all transaxial sections. Similarly, the SUV_max_ out of all slices was selected to represent the activity of the AA. To obtain a background value of FDG uptake, a ROI was placed on the RA cavity and the mean SUV (SUV_mean_) was recorded. Thereafter, a target-to-background ratio (TBR) was calculated for bilateral atria and AAs, respectively. A TBR value of AF patients beyond the mean +1.96 SD of that of control subjects was regarded as abnormally increased.

To measure the activity of EAT, tissue with Hounsfield units between -190 and -45 was defined as adipose tissue. Appropriate ROIs were placed on the adipose tissue adjacent to the origin of the right coronary artery, and the SUV_max_ was recorded.^9^ Splenic activity was obtained by placing a ROI around the spleen on all transaxial sections.^14^,^15^ The average of SUV_max_ values from all of the sections was recorded as the splenic activity. The activity of bone marrow was measured by drawing a ROI on a transaxial section of each vertebra from L-3 to L-5. The average of SUV_max_ values of the three vertebrae was calculated to determine the activity of the bone marrow.^15^

Additionally, a three-grade scoring system was employed to evaluate the adequacy of myocardial suppression (Supplementary Figure 1).^16^ The SUV_max_ of left ventricle was measured as well.

### Statistical Analysis

SPSS Statistics (Version 23; IBM) was used to perform the statistical analysis. A *P* value < 0.05 was considered to be statistically significant. Continuous variables were described as medians, with a 25% to 75% interquartile range. The normality of distribution was assessed using the Kolmogorov-Smirnoff test. Categorical variables were expressed as absolute numbers or percentages. Variables between groups were compared using the Student’s *t* test, the Mann-Whitney *U* test, the chi-square test, or the Fisher exact test where appropriate. Spearman’s correlation analysis was conducted to explore the correlations between the SUV_max_ of atrium or AA (the higher SUV_max_ of bilateral atria or AAs was selected to represent the individual patient) and related continuous variables. Intra- and inter-observer reproducibility was assessed using the intraclass correlation coefficient (ICC).

To explore the factors relevant to the increased uptake in the atrium and AA, two multivariate regression models were employed. First, all variables with a *P* value < 0.1 from the bivariate analysis were entered into a multivariate logistic regression analysis with backward elimination to investigate the relevant factors predicting the presence of increased activity in atrium or AA. Since one parameter estimate (persistent AF) diverges to infinity in the first model, Firth’s bias-adjusted estimate can guarantee the parameter estimates being finite when the sample size is small. This statistical analysis was performed using R version 3.4.3 and package Logistf.^17^,^18^ Second, continuous parameters with a *P* value < 0.1 from the bivariate analysis were entered into a multivariate linear regression analysis to detect the independent linear correlation between them and the activity in the atrium or AA.

## Results

### Patients

The demographic, clinical, and imaging parameters of the studied population are presented in Table 1. The included 48 AF patients comprised 36 persistent AF and 16 paroxysmal AF.

**Table 1:** Patients characteristics

	AF patients (*N* = 48)	Control subjects (*N* = 22)	*P* value
Male (%)	36 (75)	16 (73)	0.53
Age, years	70 (58–76)	67 (62–72)	0.92
BMI, kg/m^2^	23.2 (21.4–25.3)	22.7 (21.8–26.4)	0.21
Diabetes (%)	17(35)	8 (36)	0.75
Baseline glucose, mg/dl	98.1 (88.2–130.5)	98.1 (88.2–117.9)	0.87
Hypertension (%)	28 (58)	0 (0)	0.000
Coronary artery disease (%)	10 (21)	0 (0)	0.016
Congestive heart failure (%)	5 (10)	0 (0)	0.14
Stroke (%)	6 (13)	0 (0)	0.09
Malignancy (%)	26 (54)	0 (0)	0.000
LAVI, ml/m^2^	22.2 (14.6–34.9)	16.0 (13.5–18.0)	0.003
RA area, cm^2^	17.5 (14.7–24.3)	13.6 (12.6–14.6)	0.000
Adequate myocardial suppression	21 (44)	8 (36)	0.38
SUV_max_			
Left atrium	1.6 (1.5–1.7)	1.6 (1.3–1.8)	0.33
Right atrium	1.7 (1.5–2.6)	1.5 (1.1–1.6)	0.000
Left appendage	2.1 (1.8–2.5)	1.9 (1.5–2.1)	0.013
Right appendage	2.1 (1.9–2.6)	1.8 (1.5–2.1)	0.002
Left ventricle	3.4 (2.0–5.8)	3.0 (2.2–7.4)	0.96
Spleen	2.4 (2.1–2.7)	2.1 (1.9–2.3)	0.25
Bone marrow	2.8 (2.4–3.3)	2.4 (2.1–2.9)	0.16
Right coronary artery	1.3 (1.2–1.7)	1.0 (0.8–1.6)	0.006
SUV_mean_ of blood pool	1.8 (1.6–2.0)	1.8 (1.7–2.1)	0.56
TBR			
Left atrium	0.8 (0.8–0.9)	0.9 (0.8–0.9)	0.18
Right atrium	0.9 (0.8–1.6)	0. 8 (0.6–0.9)	0.000*
Left appendage	1.1 (1.0–1.4)	0.9 (0.8–1.1)	0.002*
Right appendage	1.2 (1.0–1.4)	0.9 (0.8–1.1)	0.000*

*AF*, atrial fibrillation; *BMI*, body mass index; *LAVI*, left atrium volume index; *RA*, right atrium; *SUV*_*max*_, maximum standardized uptake value; *SUV*_*mean*_, mean standardized uptake value; *TBR*, target-to-background ratio

No significant differences were found in demographic data (age, gender, and BMI) between the AF and control groups. More concurrent diseases other than diabetes were demonstrated in the AF group. The AF patients had larger atria bilaterally. Neither blood activity nor hematopoietic tissue activity was significantly different between the two groups. Both FDG uptake value and the adequacy of myocardial suppression in the left ventricle were not different between the AF and control groups. Of interest is that greater EAT activity was observed in the AF group.

### Atrial FDG Uptake

**Figure 2:**
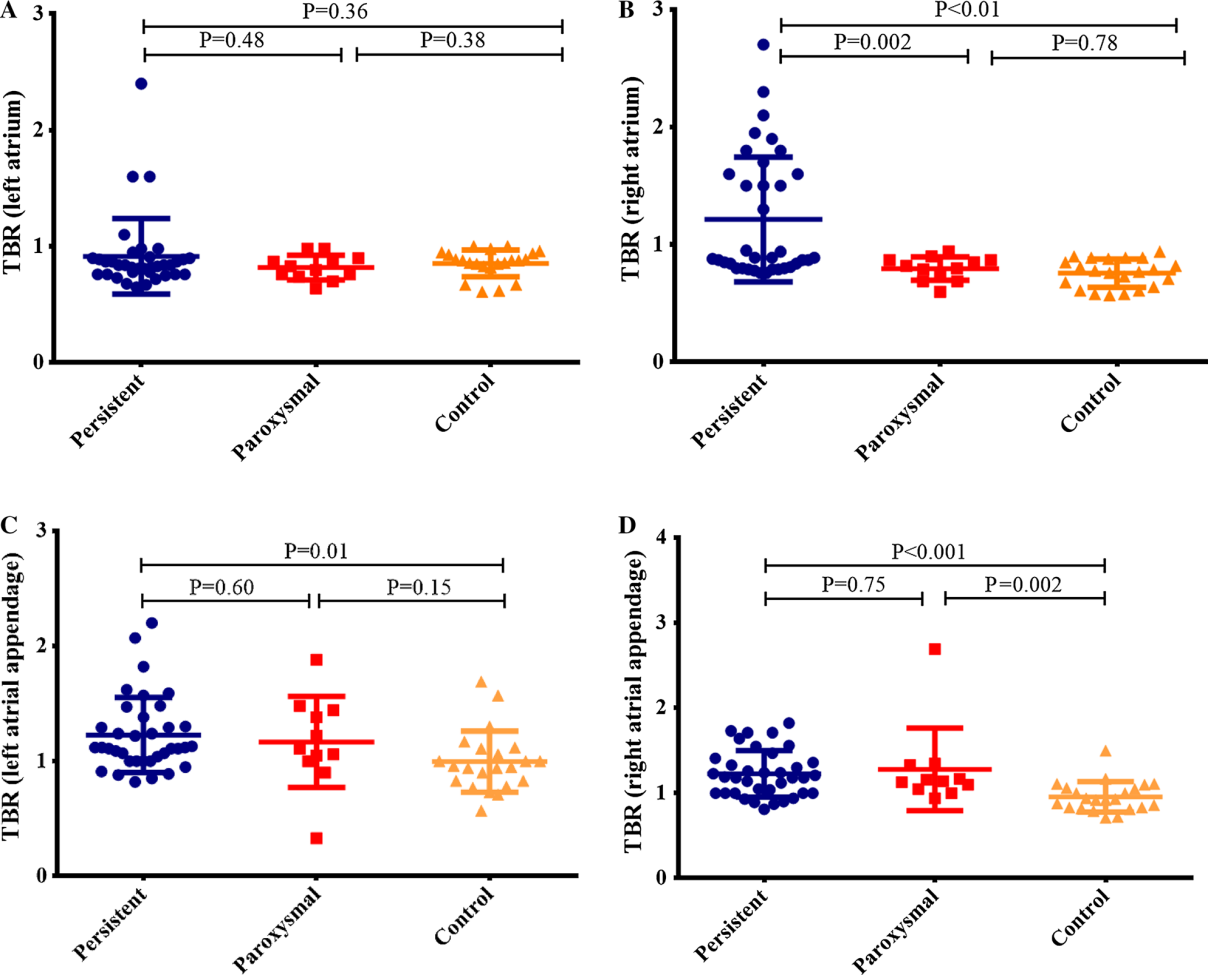
Comparisons of target-to-background ratio (TBR) values among patients with persistent atrial fibrillation (AF), paroxysmal AF, and controls (**A**, left atrium; **B**, right atrium; **C**, left atrial appendage; **D**, right atrial appendage)

**Figure 3:**
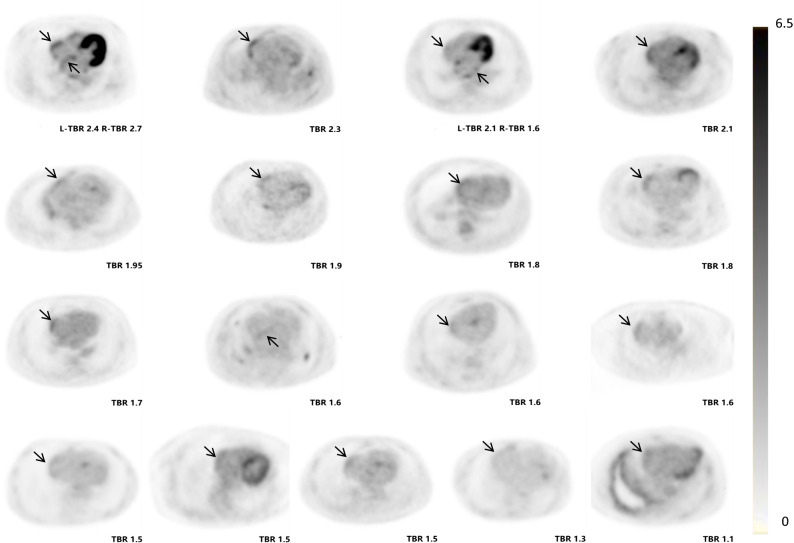
Axial positron emission tomography (PET) images of 17 atrial fibrillation (AF) patients with increased atrial uptake (arrows indicate abnormal uptake)

**Figure 4:**
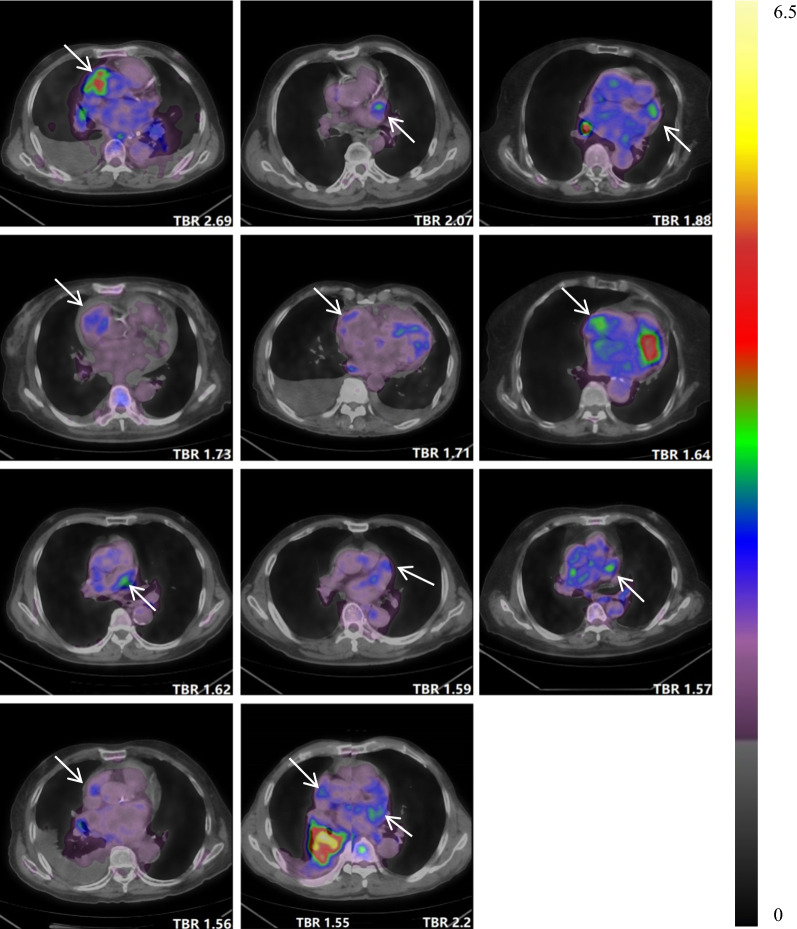
Axial positron emission tomography/computed tomography (PET/CT) images of 11 atrial fibrillation (AF) patients with increased uptake in the atrial appendage (arrows indicate abnormal uptake)

The individual SUV_max_ and TBR values of atrium and AA are presented in Supplementary Table 1. Patients in the AF group had higher FDG uptake in the RA and both AAs, whereas the uptake was not significantly different in the LA. Figure 2 illustrates the comparison of TBR values of atria and AAs in paroxysmal AF, persistent AF, and the control group. Based on the predefined definition, the cut-off values of TBR for determining increased atrial uptake were 0.95 for the LA, 0.84 for the RA, 1.23 for the left AA, and 1.12 for the right AA. As a result, 17 AF patients (35.4%) were shown to have increased atrial uptake (Figure 3). Most of them (14, or 82.4%) had abnormal uptake merely in the free wall of the RA, whereas 1 had abnormal uptake in the LA and 2 in both atria. Eleven patients (22.9%) showed increased FDG uptake in the AA (Figure 4), 5 in the left, 5 in the right, and 1 in both AAs. Of note, all patients with increased atrial uptake had persistent AF, whereas 2 of 11 patients with abnormal uptake in the AA had paroxysmal AF.

### Variables Related to FDG Uptake

Six variables (gender, age, type of AF, LAVI, RA area, and SUV_max_ of EAT) were identified from the univariate analyses to be statistically different (*P *< 0.1) between AF patients with and without increased atrial uptake (Supplementary Table 2). Multivariate logistic regression analyses demonstrated that three factors (female gender, persistent AF, and EAT activity) independently predicted the increased activity in atria (Table 2). As to AAs, five variables (congestive heart failure, LAVI, RA area, SUV_max_ of LV, and SUV_max_ of EAT) were statistically different (*P* < 0.1) in univariate analysis (Supplementary Table 3), and one factor (SUV_max_ of the LA) remained significant after multivariate regression analyses (Table 2).

**Table 2:** Multivariate logistic regression analyses of variables predicting the enhanced activity in atrium and atrial appendage

Variable	Odds ratio	95% CI	*P* value
Atrium			
Male	− 2.42	− 20.82 to − 4.48	0.02*
Age, years	0.08	− 0.01 to 0.19	0.07
Persistent AF	3.31	0.89 to 8.31	< 0.001*
LAVI, ml/m^2^	− 0.08	− 0.19 to 0.00	0.06
RA area, cm^2^	0.15	− 0.07 to 0.44	0.18
SUV_max_ of RCA	2.47	0.16 to 5.33	0.04*
Atrial appendage			
Congestive heart failure	5.55	0.29 to 41.5	0.323
LAVI, ml/m^2^	0.95	0.87 to 1.04	0.265
RA area, cm^2^	1.09	0.91 to 1.15	0.674
SUV_max_ of LV	1.19	1.01 to 1.41	0.002*
UV_max_ of RCA	2.14	0.14 to 32.0	0.579

*LAVI*, left atrium volume index; *RA*, right atrium; *SUV*_*max*_, maximum standardized uptake value; *LV*, left ventricle; *RCA*, right coronary artery

**Figure 5:**
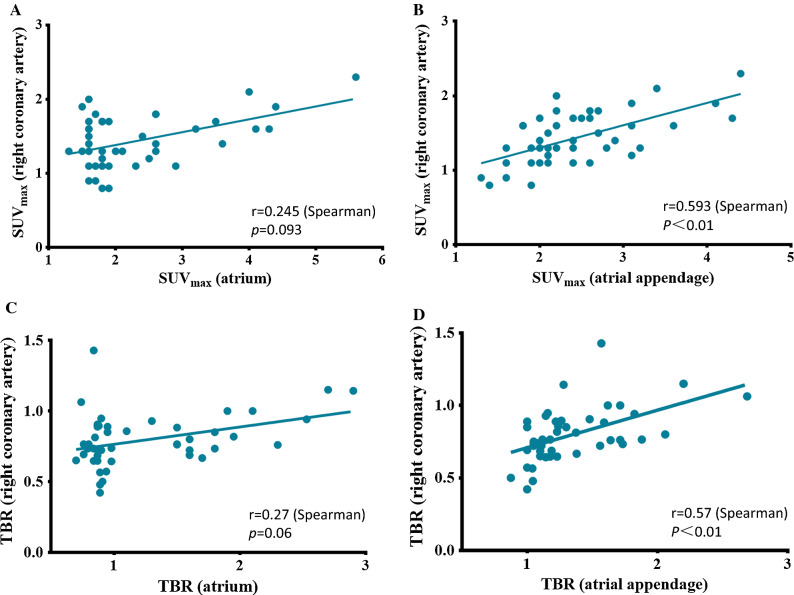
Linear correlation analysis between the maximum standardized uptake value, (SUVmax) of epicardial adipose tissue (EAT) in right coronary artery and that of atrium (**A**) and atrial appendage (**B**). Linear correlation analysis between the TBR of EAT in the right coronary artery and the TBR of atrium (**C**) and atrial appendage (**D**)

Multivariate linear regression analyses demonstrated that SUV_max_ of EAT was the only factor independently associated with the activity of the atrium and AA (Table 3 and Figure 5).

**Table 3:** Univariate and multivariate linear regression analyses of variables correlated with the activity in atrium and atrial appendage

	Univariate linear regression		Multivariate linear regression			
	*r*	*P* value	B ± SE	(95% CI)	*P* value	Adjusted R^2^
Atrium						0.225
Age, years	0.112	0.44	0.015 ± 0.013	− 0.011 to 0.041	0.255	
LAVI, ml/m^2^	0.191	0.19	− 0.017± 0.014	− 0.046 to 0.012	0.238	
RA area, cm^2^	0.163	0.26	0.038 ± 0.036	− 0.035 to 0.112	0.296	
SUV_max_ of RCA	0.245	0.093	1.39 ± 0.375	0.635 to 2.146	< 0.001	
Atrial appendage						0.387
LAVI, ml/m^2^	0.336	0.02	− 0.01 ± 0.01	− 0.029 to 0.009	0.29	
RA area, cm^2^	0.279	0.06	0.036 ± 0.024	0.012 to 0.083	0.13	
SUV_max_ of LV	0.384	0.007	0.015 ± 0.024	− 0.033 to 0.063	0.54	
SUV_max_ of RCA	0.593	0.000	1.183 ± 0.295	0.587 to 1.778	< 0.001	

*LAVI*, left atrium volume index; *RA*, right atrium; *SUV*_*max*_, maximum standardized uptake value; *LV*, left ventricle; *RCA*, right coronary artery

### Reproducibility

Supplementary Table 4 presents the reproducibility of FDG uptake measurements. Both intra- and inter-observer comparisons showed excellent reproducibility in all of the measurements (all ICC > 0.8).

## Discussion

The present study first explored the factors relevant to the atrial FDG uptake in patients with AF. Although the pathologic mechanism could not be clarified by this study, the characteristics of patients with abnormal FDG uptake and several factors related to the abnormal uptake were detected. First, atrial uptake was observed mainly in persistent AF and in the RA, whereas bilateral AAs could be equally involved. Second, the activity of EAT was independently correlated with the activity of the atrium and AA.

### Characteristics of Atrial FDG Uptake

Several characteristics of atrial FDG uptake in AF were found. Of interest, we found that abnormal atrial uptake existed only in persistent AF, suggesting that AF induces atrial activity rather than the opposite. In comparing persistent AF with paroxysmal AF, electroanatomic mapping and histologic studies showed that patients with persistent AF had larger LAs, lower atrial voltage, slower atrial conduction, shorter cycle length, and greater atrial fibrosis than those with paroxysmal AF.^19^ Presumably, the abnormal atrial FDG uptake reflects a certain pattern of abnormal atrial substrate during the progression of AF.

Another characteristic was that most of the atrial uptake took place in the RA; the LA was rarely involved. It is well known that the LA and left AA are predominantly responsible for the initiation of fibrillary activity and thrombosis, which is rare in the RA and right AA. The specific reason for this is unclear. Observational studies on AF have demonstrated that the LA has a more extensive matrix and subendocardial remodeling than the RA,^20^,^21^ suggesting that the RA is better protected than the LA. Accordingly, the accumulation of FDG mainly in the RA may be explained by the advanced fibrotic burden in the LA, which cannot extract FDG.

Furthermore, this study found that the distribution pattern and relevant factors were different in the atrium and AA, which indicated that they may have different pathologic mechanisms and clinical significance. The AA is prone to thrombosis and is closely involved in stroke and other thromboembolic events;^22^ therefore, the prognostic value of FDG uptake in the atrial wall and AA needs to be separately considered in the future studies.

### Inflammation and Atrial Activity

Recent advances have revealed that inflammation is related to the initiation and persistence of AF.^6^–^8^ Activated inflammatory cells have been shown to take in more glucose; in particular, physiologic uptake within the myocardium was largely suppressed under fasting conditions. Therefore, atrial FDG uptake in AF patients may be attributed to the enhanced infiltration of inflammatory cells. Our patients were instructed to maintain a prolonged fast of at least 12 hours prior to the administration of FDG. A prolonged fast has been reported to be more effective in suppressing physiologic uptake.^23^,^24^ However, whether the atrial FDG signals observed were localized to the inflammatory cells requires further pathologic study.

A novel finding in our study was that the activity of EAT was linearly correlated with the activity of the atrium and AA. There is no fascial boundary between the EAT and atrial myocardium, and the EAT contains numerous autonomic ganglion and progenitor cells.^25^ The EAT was therefore the source of inflammatory cytokines and myofibroblasts, which produced the extracellular matrix. Previous studies have shown that patients with AF had a larger and deeper EAT compared with patients without AF.^26^–^28^ Using PET/CT, Mazurek et al. reported that AF individuals had higher FDG activity in the EAT than non-AF individuals.^9^ However, the relationship between atrial activity and EAT activity has not been previously reported. Similar to Mazurek’s study, we also found increased activity in the EAT and further demonstrated that this was correlated with the atrial activity. This finding suggests a link between the activity of the EAT and atrium in AF, pointing to the possibility that a local inflammatory burden in the EAT may lead to localized atrial inflammation.

Joseph et al. have reported that as the “incubator” for monocytes, the activity of hematopoietic tissue (spleen) is more elevated in AF participants than in controls.^10^ However, the difference in splenic activity did not reach significance between AFs and controls in our study. Moreover, many studies have reported higher levels of inflammatory markers (C-reactive protein) in the systemic circulation in AF subjects.^29^–^31^ Regrettably, C-reactive protein was measured in only some of the patients in our study, and we found no linear correlations between the level of C-reactive protein and the SUV_max_ of atria or AAs (Supplementary Figure 2). The discrepancies in the analyses of hematopoietic tissues and circulation markers between the present and prior studies warrant further exploration.

### Other Factors Related to Atrial Activity

In the present study, patients with AF had larger atria than controls; more importantly, patients with increased activity in the atrium and AA had even larger atria than those without. Atrial size is correlated with the atrial pressure,^32^ which may induce enhanced atrial FDG uptake.^33^ However, consistent with Fujii’s observation, although the LA was dilated as well, abnormal FDG uptake was located mainly in the RA. This result supports the multiple mechanisms of atrial FDG uptake in AF.

Previous studies have revealed that atrial ischemia can induce atrial fibrillation and vice versa.^34^ Whether ischemia contributes to increased atrial FDG uptake cannot be determined from the current study. Further study to clarify the relationship between ischemia and atrial FDG uptake in AF is needed.

This study observed a relationship between LV FDG uptake and the activity of AA. However, the LV uptake is heterogeneous under conventional fasting conditions, and physiological uptake can not be excluded. Therefore, this issue need to be further studied employing effective strategies to suppress myocardial physiological uptake.

### Limitations

Restricted to its retrospective nature and small size, this study has several limitations. First, we cannot determine the pathologic or histologic properties of abnormal FDG uptake in the atrium. Second, the enrolled patients were selected from among those who underwent a whole-body PET imaging procedure mainly for oncologic evaluation. Although the demographic characteristics and concurrent diseases were comparable between patients with and without atrial uptake, etiologies other than AF that may result in abnormal atrial FDG uptake could not be excluded.^35^ Third, although a prolonged fast (>12 hours) was employed in our imaging protocol, physiologic FDG uptake in atrial myocardium may not be suppressed completely. Numerous studies have analyzed the characteristics of myocardial FDG uptake in routine FDG/PET imaging and have explored more efficient strategies to suppress physiologic uptake for inflammatory or ischemic imaging;^36^ however, these had focused only on the left ventricle. An appropriate imaging strategy for atrial FDG imaging is not readily available. Fourth, in this cross-sectional study, the causal relationship between atrial FDG uptake and AF could not be explored. To elucidate these issues, a prospective study exploring the methodology, pathologic features, and clinical significance of atrial FDG uptake in AF patients is under way at our institution.

## Conclusions

This study demonstrates that enhanced uptake of FDG in the atria is present in persistent AF, localized mainly in the RA, whereas bilateral AAs could be equally involved. Multiple factors contribute to the increased atrial activity, and the EAT activity was independently correlated with the activity of the atria and AAs. Since inflammation has been considered an important mechanism in the genesis and perpetuation of AF, further studies exploring the pathologic features and clinical significance of elevated atrial activity are warranted.

## New knowledge Gained

Increased activity of EAT, a marker of localized inflammation, was independently associated with the enhanced FDG uptake in atrial wall and AA in AF patients.

## Electronic supplementary material

Below is the link to the electronic supplementary material.

Supplementary material 1 (DOC 323 kb)

Supplementary material 1 (PPTX 5916 kb)

## Abbreviations

FDG: Fluorodeoxyglucose
AF: Atrial fibrillation
PET/CT: Positron emission tomography/computerized tomography
SUV: Standardized uptake value
TBR: Target-to-background ratio
AA: Atrial appendage
EAT: Epicardial adipose tissue
LAVI: Left atrium volume index
LA: Left atrium
RA: Right atrium
ROI: Region of interest

## References

[1] Fujii H, Yasuda S, Ide M, Takahashi W, Shohtsu A, Kubo A (1999). Increased fluorine-18 fluorodeoxyglucose uptake in the right atrial wall in a patient with atrial fibrillation. Clin Nucl Med.

[2] Fujii H, Ide M, Yasuda S, Takahashi W, Shohtsu A, Kubo A (1999). Increased FDG uptake in the wall of the right atrium in people who participated in a cancer screening program with whole-body PET. Ann Nucl Med.

[3] Nguyen BD (2005). PET demonstration of left atrial appendage in chronic atrial fibrillation. Clin Nucl Med.

[4] Okura K, Maeno K, Hirazawa M, Takemori H, Toya D, Tanaka N (2012). Fluorodeoxyglucose accumulation in the left atrial appendage of a patient with paroxysmal atrial fibrillation. J Cardiol Cases.

[5] Dong A, Zhao T, Gong J, Zuo C (2014). Diffuse FDG uptake of the bilateral atrial walls in a patient with atrial fibrillation. Clin Nucl Med.

[6] Aviles RJ, Martin DO, Apperson-Hansen C, Houghtaling PL, Rautaharju P, Kronmal RA (2003). Inflammation as a risk factor for atrial fibrillation. Circulation.

[7] Yamashita T, Sekiguchi A, Iwasaki YK, Date T, Sagara K, Tanabe H (2010). Recruitment of immune cells across atrial endocardium in human atrial fibrillation. Circ J.

[8] Frustaci A, Chimenti C, Bellocci F, Morgante E, Russo MA, Maseri A (1997). Histological substrate of atrial biopsies in patients with lone atrial fibrillation. Circulation.

[9] Mazurek T, Kiliszek M, Kobylecka M, Skubisz-Głuchowska J, Kochman J, Filipiak K (2014). Relation of proinflammatory activity of epicardial adipose tissue to the occurrence of atrial fibrillation. Am J Cardiol.

[10] Joseph P, Ishai A, MacNabb M, Abdelbaky A, Lavender ZR, Ruskin J (2016). Atrial fibrillation is associated with hematopoietic tissue activation and arterial inflammation. Int J Cardiovasc Imaging.

[11] James OG, Christensen JD, Wong TZ, Borges-Neto S, Koweek LM (2011). Utility of FDG PET/CT in inflammatory cardiovascular disease. Radiographics.

[12] Abhayaratna WP, Seward JB, Appleton CP, Douglas PS, Oh JK, Tajik AJ (2006). Left atrial size: physiologic determinants and clinical applications. J Am Coll Cardiol.

[13] Grünig E, Henn P, D’Andrea A, Claussen M, Ehlken N, Maier F (2013). Reference values for and determinants of right atrial area in healthy adults by 2-dimensional echocardiography. Circ Cardiovasc Imaging.

[14] Kim EJ, Kim S, Kang DO, Seo HS (2014). Metabolic activity of the spleen and bone marrow in patients with acute myocardial infarction evaluated by 18f-fluorodeoxyglucose positron emission tomographic imaging. Circ Cardiovasc Imaging.

[15] Emami H, Singh P, MacNabb M, Vucic E, Lavender Z, Rudd JH (2015). Splenic metabolic activity predicts risk of future cardiovascular events: demonstration of a cardiosplenic axis in humans. JACC Cardiovasc Imaging.

[16] Osborne MT, Hulten EA, Murthy VL (2017). Patient preparation for cardiac fluorine-18 fluorodeoxyglucose positron emission tomography imaging of inflammation. J Nucl Cardiol.

[17] Firth D (1993). Bias reduction of maximum likelihood estimates. Biometrika.

[18] Team RDC (2016). R: A language and environment for statistical computing. R foundation for statistical computing, Vienna, Austria. Computing.

[19] Lau DH, Linz D, Schotten U, Mahajan R, Sanders P, Kalman JM (2017). Pathophysiology of paroxysmal and persistent atrial fibrillation: Rotors. Foci and Fibrosis. Heart Lung Circ..

[20] Margulescu AD, Mont L (2017). Persistent atrial fibrillation vs paroxysmal atrial fibrillation: Differences in management. Expert Rev Cardiovasc Ther.

[21] Park JH, Lee JS, Ko YG, Lee SH, Lee BS, Kang SM (2014). Histological and biochemical comparisons between right atrium and left atrium in patients with mitral valvular atrial fibrillation. Korean Circ J.

[22] Modrego J, Maroto L, Tamargo J, Azcona L, Mateos-Cáceres P, Segura A (2010). Comparative expression of proteins in left and right atrial appendages from patients with mitral valve disease at sinus rhythm and atrial fibrillation. J Cardiovasc Electrophysiol.

[23] Manabe O, Yoshinaga K, Ohira H, Masuda A, Sato T, Tsujino I (2016). The effects of 18-h fasting with low-carbohydrate diet preparation on suppressed physiological myocardial (18)F-fluorodeoxyglucose (FDG) uptake and possible minimal effects of unfractionated heparin use in patients with suspected cardiac involvement sarcoidosis. J Nucl Cardiol.

[24] Lu Y, Grant C, Xie K, Sweiss NJ (2017). Suppression of myocardial ^18^F-FDG uptake through prolonged high-Fat, high-protein, and very-low-carbohydrate diet before FDG-PET/CT for evaluation of patients with suspected cardiac sarcoidosis. Clin Nucl Med.

[25] Mazurek T, Zhang L, Zalewski A, Mannion JD, Diehl JT, Arafat H (2003). Human epicardial adipose tissue is a source of inflammatory mediators. Circulation.

[26] van Rosendael AR, Dimitriu-Leen AC, van Rosendael PJ, Leung M, Smit JM, Saraste A (2017). Association between posterior left atrial adipose tissue mass and atrial fibrillation. Circ Arrhythm Electrophysiol.

[27] Sacks HS, Fain JN (2007). Human epicardial adipose tissue: a review. Am Heart J.

[28] Hatem SN, Sanders P (2014). Epicardial adipose tissue and atrial fibrillation. Cardiovasc Res.

[29] Hu YF, Chen YJ, Lin YJ, Chen SA (2015). Inflammation and the pathogenesis of atrial fibrillation. Nat Rev Cardiol.

[30] Liuba I, Ahlmroth H, Jonasson L, Englund A, Jönsson A, Säfström K (2008). Source of inflammatory markers in patients with atrial fibrillation. Europace.

[31] Marcus GM, Smith LM, Ordovas K, Scheinman MM, Kim AM, Badhwar N (2010). Intracardiac and extracardiac markers of inflammation during atrial fibrillation. Heart Rhythm.

[32] Gerdts E, Oikarinen L, Palmieri V, Otterstad JE, Wachtell K, Boman K (2002). Correlates of left atrial size in hypertensive patients with left ventricular hypertrophy: The losartan intervention for endpoint reduction in hypertension (LIFE) study. Hypertension.

[33] Hagan G, Southwood M, Treacy C, Ross RM, Soon E, Coulson J (2011). (18)FDG PET imaging can quantify increased cellular metabolism in pulmonary arterial hypertension: A proof-of-principle study. Pulm Circ.

[34] Sinno H, Derakhchan K, Libersan D, Merhi Y, Leung TK, Nattel S (2003). Atrial ischemia promotes atrial fibrillation in dogs. Circulation.

[35] Lange PS, Avramovic N, Frommeyer G, Wasmer K, Pott C, Eckardt L (2017). Routine ^18^F-FDG PET/CT does not detect inflammation in the left atrium in patients with atrial fibrillation. Int J Cardiovasc Imaging.

[36] Scholtens AM, Verberne HJ, Budde RP, Lam MG (2016). Additional heparin preadministration improves cardiac glucose metabolism suppression over low-carbohydrate diet alone in ^18^F-FDG PET Imaging. J Nucl Med.

