# Supplementary material for: Factors relevant to atrial 18F-fluorodeoxyglucose uptake in atrial fibrillation
Source: J Nucl Cardiol. 2018 Aug 7;27(5):1501–12. doi: 10.1007/s12350-018-1387-4 (PMC7599132; doi:10.1007/s12350-018-1387-4)

**Supplemental Table 1. FDG uptake values in atrium and AA**

| **Patient no.** | **Group (persist, paroxysmal, control)** | **Gender** | **Age (y)** | **LA** | | **RA** | |
| --- | --- | --- | --- | --- | --- | --- | --- |
|  |  |  |  | SUV_max_ | TBR | SUV_max_ | TBR |
| **1** | Persistent | Male | 51 | 1.6 | 0.84 | 1.5 | 0.95 |
| **2** | Persistent | Male | 61 | 1.8 | 0.72 | 2.1 | 1.5 |
| **3** | Persistent | Female | 65 | 2.0 | 0.78 | 2.6 | 1.1 |
| **4** | Persistent | male | 53 | 1.3 | 0.81 | 2.0 | 0.87 |
| **5** | Persistent | Female | 77 | 1.7 | 0.82 | 3.2 | 1.5 |
| **6** | Persistent | Male | 56 | 1.6 | 0.73 | 1.5 | 0.84 |
| **7** | Persistent | Male | 77 | 2.6 | 1.6 | 1.3 | 0.8 |
| **8** | Persistent | Female | 73 | 5.1 | 2.4 | 5.6 | 2.7 |
| **9** | Persistent | Male | 58 | 1.5 | 0.84 | 1.8 | 0.88 |
| **10** | Persistent | Male | 72 | 1.6 | 0.81 | 1.5 | 0.79 |
| **11** | Persistent | Female | 55 | 1.5 | 0.76 | 1.8 | 0.89 |
| **12** | Persistent | Male | 56 | 1.6 | 0.84 | 1.8 | 0.76 |
| **13** | Persistent | Male | 74 | 1.4 | 0.76 | 1.6 | 0.8 |
| **14** | Persistent | Male | 68 | 1.9 | 0.91 | 1.6 | 0.89 |
| **15** | Persistent | Male | 65 | 3.1 | 1.6 | 4.0 | 2.1 |
| **16** | Persistent | Male | 76 | 1.7 | 0.89 | 1.6 | 0.85 |
| **17** | Persistent | Male | 60 | 1.6 | 0.76 | 1.4 | 0.79 |
| **18** | Persistent | Male | 81 | 1.5 | 0.82 | 1.6 | 0.84 |
| **19** | Persistent | Male | 76 | 1.8 | 0.76 | 1.7 | 0.94 |
| **20** | Persistent | Male | 67 | 1.5 | 0.67 | 2.4 | 1.5 |
| **21** | Persistent | Male | 77 | 1.6 | 0.87 | 3.5 | 1.8 |
| **22** | Persistent | Male | 55 | 1.5 | 0.65 | 1.6 | 0.76 |
| **23** | Persistent | Male | 77 | 1.3 | 0.89 | 1.5 | 0.87 |
| **24** | Persistent | Male | 75 | 1.4 | 0.75 | 4.4 | 2.3 |
| **25** | Persistent | Female | 67 | 1.7 | 0.78 | 4.3 | 2.53 |
| **26** | Persistent | Male | 59 | 1.6 | 0.84 | 2.5 | 1.6 |
| **27** | Persistent | Female | 56 | 1.5 | 0.87 | 1.9 | 0.81 |
| **28** | Persistent | Male | 50 | 1.3 | 0.9 | 1.3 | 0.8 |
| **29** | Persistent | Female | 83 | 1.8 | 0.95 | 1.6 | 0.87 |
| **30** | Persistent | Male | 71 | 1.7 | 0.98 | 2.6 | 1.95 |
| **31** | Persistent | Female | 76 | 1.6 | 0.83 | 1.6 | 1.3 |
| **32** | Persistent | Male | 90 | 1.6 | 0.98 | 2.6 | 1.9 |
| **33** | Persistent | Female | 79 | 1.5 | 0.9 | 2.3 | 1.6 |
| **34** | Persistent | Female | 66 | 1.6 | 0.68 | 2.9 | 1.8 |
| **35** | Persistent | Male | 77 | 1.5 | 0.87 | 3.6 | 1.7 |
| **36** | Persistent | Male | 58 | 1.5 | 0.78 | 4.1 | 2.9 |
| **37** | Persistent | Male | 53 | 1.7 | 0.74 | 1.9 | 0.69 |
| **38** | Paroxysmal | Male | 73 | 1.5 | 0.8 | 1.4 | 0.87 |
| **39** | Paroxysmal | Male | 79 | 1.8 | 0.7 | 1.4 | 0.6 |
| **40** | Paroxysmal | Male | 63 | 1.3 | 0.87 | 1.6 | 0.75 |
| **41** | Paroxysmal | Male | 52 | 2.0 | 0.64 | 1.5 | 0.79 |
| **42** | Paroxysmal | Female | 70 | 1.7 | 0.98 | 1.3 | 0.9 |
| **43** | Paroxysmal | Male | 69 | 1.9 | 0.87 | 1.5 | 0.82 |
| **44** | Paroxysmal | Male | 79 | 1.6 | 0.83 | 1.5 | 0.85 |
| **45** | Paroxysmal | Male | 53 | 1.7 | 0.9 | 1.3 | 0.87 |
| **46** | Paroxysmal | Male | 75 | 1.5 | 0.76 | 1.6 | 0.8 |
| **47** | Paroxysmal | Male | 73 | 2.0 | 0.98 | 1.9 | 0.94 |
| **48** | Paroxysmal | Female | 73 | 1.4 | 0.76 | 1.5 | 0.69 |
| **49** | Control | Female | 76 | 1.0 | 0.98 | 0.8 | 0.78 |
| **50** | Control | Female | 71 | 1.9 | 1.00 | 1.3 | 0.68 |
| **51** | Control | Female | 74 | 1.7 | 0.85 | 1.8 | 0.90 |
| **52** | Control | Female | 71 | 2.1 | 0.88 | 1.7 | 0.71 |
| **53** | Control | Female | 77 | 1.6 | 0.89 | 1.1 | 0.61 |
| **54** | Control | Female | 77 | 1.6 | 0.89 | 1.1 | 0.61 |
| **55** | Control | Male | 60 | 1.8 | 0.86 | 1.2 | 0.57 |
| **56** | Control | Male | 67 | 1.2 | 0.67 | 1.7 | 0.94 |
| **57** | Control | Male | 66 | 1.5 | 0.83 | 1.6 | 0.89 |
| **58** | Control | Male | 65 | 1.3 | 0.62 | 1.7 | 0.81 |
| **59** | Control | Male | 61 | 1.4 | 0.82 | 1.5 | 0.88 |
| **60** | Control | Male | 60 | 1.1 | 0.61 | 1.6 | 0.89 |
| **61** | Control | Male | 60 | 1.2 | 0.67 | 1.6 | 0.89 |
| **62** | Control | Male | 60 | 1.6 | 0.94 | 1.4 | 0.82 |
| **63** | Control | Male | 78 | 1.5 | 0.88 | 1.3 | 0.76 |
| **64** | Control | Male | 64 | 1.4 | 0.93 | 1.1 | 0.73 |
| **65** | Control | Male | 69 | 1.2 | 0.86 | 0.9 | 0.64 |
| **66** | Control | Male | 62 | 1.3 | 1.0 | 1.0 | 0.77 |
| **67** | Control | Male | 66 | 1.8 | 0.95 | 1.5 | 0.79 |
| **68** | Control | Male | 67 | 2.5 | 0.96 | 1.5 | 0.58 |
| **69** | Control | Male | 64 | 2.2 | 0.85 | 2.2 | 0.85 |
| **70** | Control | Male | 69 | 2.1 | 0.88 | 1.4 | 0.58 |

**Supplemental Table 2. Univariable comparison between AF patients with- and without increased atrial uptake**

|  | With increased atrial uptake  (n=17) | Without increased atrial uptake  (n=31) | *P* value |
| --- | --- | --- | --- |
| Male (%) | 10（59） | 26（84） | 0.08 |
| Age, years | 73（66–77） | 68（55–75） | 0.05 |
| BMI, kg/m^2^ | 22.7（21.6–23.6） | 23.4（21.3–27.0） | 0.36 |
| Persistent AF | 17 (100) | 19 (61) | 0.004* |
| Duration for AF diagnosis, months | 24（1–66） | 6（1–60） | 0.78 |
| History of radiofrequency ablation | 0（0） | 2（6） | 0.53 |
| Diabetes (%) | 4（24） | 13（42） | 0.34 |
| Baseline glucose, mg/dl | 97.2 (84.6–124.2) | 106.2 (88.2–127.8) | 0.40 |
| Hypertension (%) | 11（65） | 17（55） | 0.55 |
| Coronary artery disease (%) | 5（29） | 5（16） | 0.29 |
| Congestive heart failure (%) | 3（18） | 2（6） | 0.33 |
| Stroke (%) | 3（18） | 3（10） | 0.65 |
| Malignancies (%) | 9（53） | 17（55） | 0.35 |
| LAVI, ml/m^2^ | 32.6（22.0–38.2） | 16.7（13.7–22.5） | 0.001* |
| RA area, cm^2^ | 23.0（17.3–26.8） | 16.1（13.2–18.0） | 0.002* |
| Adequate myocardial suppression | 10 (59) | 11 (35) | 0.11 |
| SUV_max_ |  |  |  |
| Left ventricle | 2.8（2.1–5.5） | 4.0（2.0–5.9） | 0.72 |
| Spleen | 2.2（2.0–2.9） | 2.4（2.1–2.6） | 0.93 |
| Bone marrow | 2.6（2.0–3.2） | 2.8（2.5–3.4） | 0.13 |
| Right coronary artery | 1.5（1.3–1.8） | 1.3（1.1–1.6） | 0.05 |

AF, atrial fibrillation; BMI, body mass index; LAVI, left atrium volume index; RA, right atrium; SUV_max_, maximum standardized uptake value; SUV_mean_, mean standardized uptake value.

**Supplemental Table 3. Univariable comparison between AF patients with- and without increased uptake in atrial appendage**

|  | With increased appendage uptake  (n=11) | Without increased appendage uptake  (n=37) | *P* value |
| --- | --- | --- | --- |
| Male (%) | 8（73） | 28（76） | 0.56 |
| Age, years | 67（59-75） | 70（57-77） | 0.99 |
| BMI, kg/m^2^ | 23.1（21.5-23.5） | 22.7（21.3-26.0） | 0.48 |
| Persistent AF (%) | 9（82） | 27（73） | 0.43 |
| Duration for AF diagnosis, months | 2（1-96） | 6（1-48） | 0.81 |
| History of radiofrequency ablation | 0（0） | 2（5） | 1.0 |
| Diabetes (%) | 3（27） | 14（38） | 0.72 |
| Baseline glucose, mg/dl | 99.0 (88.2–144.0) | 97.2 (79.1–123.3) | 0.76 |
| Hypertension (%) | 8（73） | 20（54） | 0.32 |
| Coronary artery disease (%) | 4（36） | 6（16） | 0.21 |
| Congestive heart failure (%) | 3（27） | 2（5） | 0.07 |
| Stroke (%) | 0（0） | 6（16） | 0.19 |
| Malignancies (%) | 4（36） | 22（59） | 0.16 |
| LAVI, ml/m^2^ | 34.3（21.6-39.6） | 21.2（13.7-31.6） | 0.029* |
| RA area, cm^2^ | 25.4（19.7-29.3） | 16.6（13.8-20.8） | 0.008* |
| Adequate myocardial suppression | 4 (36) | 17 (46) | 0.42 |
| SUV_max_ |  |  |  |
| Left ventricle | 5.1（2.8–11.3） | 3.0（2.0–5.0） | 0.05 |
| Spleen | 2.2（2.1-3.0） | 2.4（2.1-2.7） | 0.96 |
| Bone marrow | 2.6（2.2-3.1） | 2.8（2.4-3.5） | 0.35 |
| Right coronary artery | 1.5（1.3-2.0） | 1.3（1.1-1.6） | 0.035* |

AF, atrial fibrillation; BMI, body mass index; LAVI, left atrium volume index; RA, right atrium; SUV_max_, maximum standardized uptake value; SUV_mean_, mean standardized uptake value.

**Suppl. Table 4 Inter- and intra-observer reproducibility of SUV measurements**

| Parameters | Inter-observer ICC | Intra-observer ICC |
| --- | --- | --- |
| Left atrium | 0.86* | 0.81* |
| Right atrium | 0.98* | 095* |
| Left appendage | 0.94* | 0.93* |
| Right appendage | 0.99* | 0.96* |
| Spleen | 0.95* | 0.91* |
| Bone marrow | 0.96* | 0.93* |
| Right coronary artery | 0.93* | 0.85* |
| Blood pool | 0.93* | 0.95* |

SUV, standadiazed uptake value; ICC,intraclass correlation coefficient.

**P*<0.001.

**Suppl. Figure 1 Illustration of assessing myocardial suppression.**


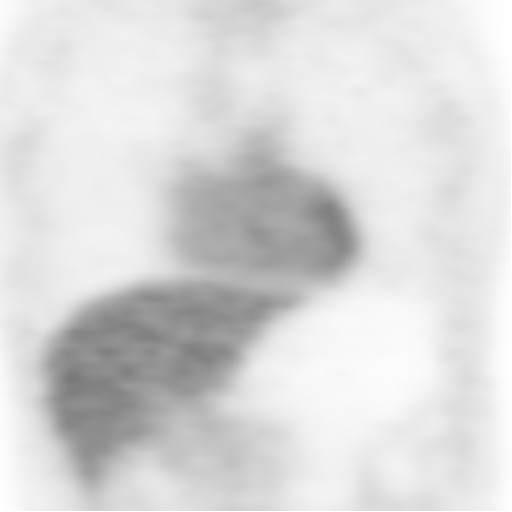


**B**


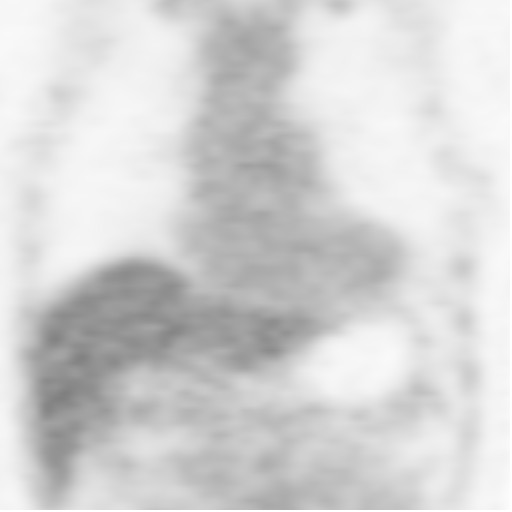


**A**


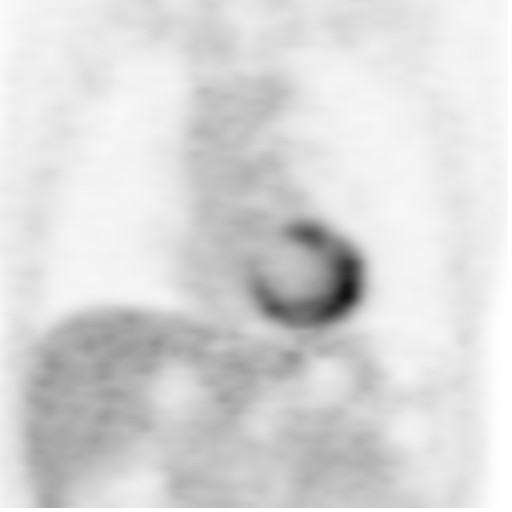


**C**

**Grade A**, excellent myocardial suppression with myocardial uptake that lower than blood-pool activity; **Grade B**, moderate myocardial suppression with diffuse low-level myocardial FDG uptake and nonspecific focally increased uptake in papillary muscles and lateral wall; **Grade C**, poor myocardial suppression with regional or diffuse high-level FDG uptake. Grade A was thought to be adequately suppressed.

**Suppl. Figure 2：There was no linear correlation between CRP and SUV_max_ of atrium (A) or atrial appendage (B).**


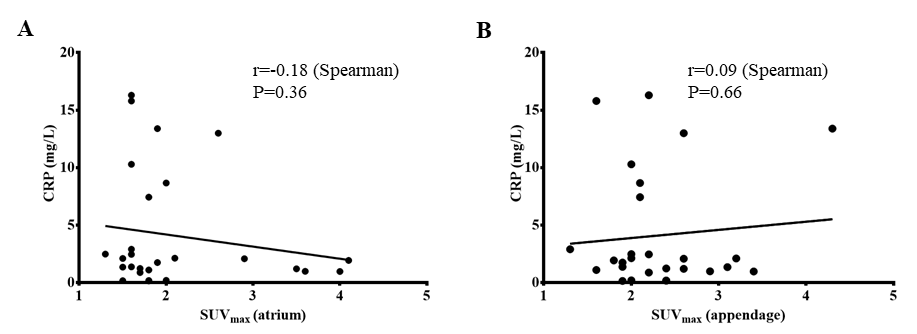

Supplement: Supplementary file 1 — Supplementary material 1 (DOC 323 kb) [file 12350_2018_1387_MOESM1_ESM.doc]
