# Supplementary material for: Factors relevant to atrial 18F-fluorodeoxyglucose uptake in atrial fibrillation
Source: J Nucl Cardiol. 2018 Aug 7;27(5):1501–12. doi: 10.1007/s12350-018-1387-4 (PMC7599132; doi:10.1007/s12350-018-1387-4)
Supplement: Supplementary file 2 — Supplementary material 1 (PPTX 5916 kb) [file 12350_2018_1387_MOESM2_ESM.pptx]

## Slide 1
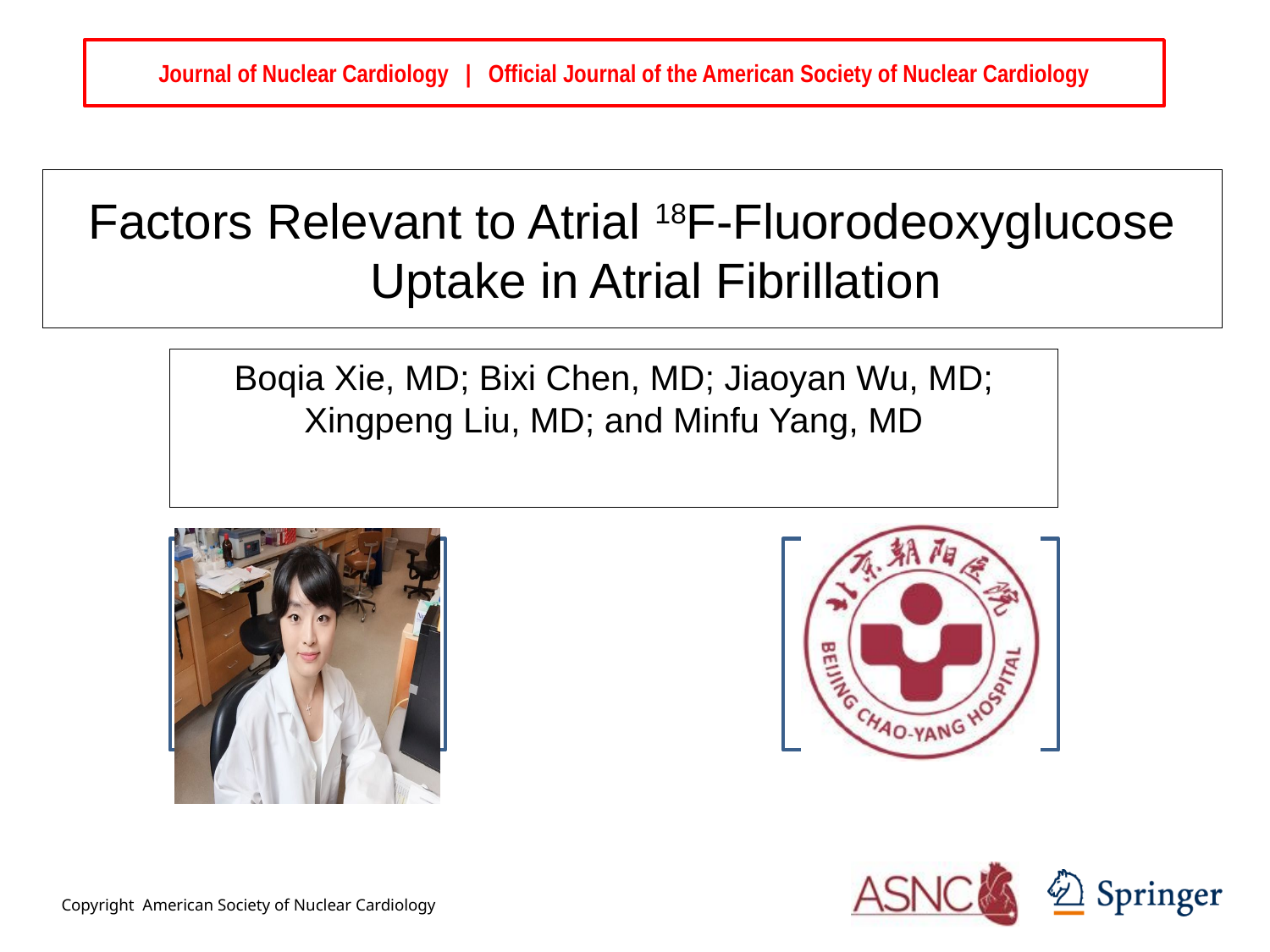

Journal of Nuclear Cardiology | Official Journal of the American Society of Nuclear Cardiology
# Factors Relevant to Atrial 18F-Fluorodeoxyglucose Uptake in Atrial Fibrillation
Boqia Xie, MD; Bixi Chen, MD; Jiaoyan Wu, MD; Xingpeng Liu, MD; and Minfu Yang, MD
Head shot of author
required
Institution
Picture/Logo
Optional
Copyright American Society of Nuclear Cardiology

## Slide 2
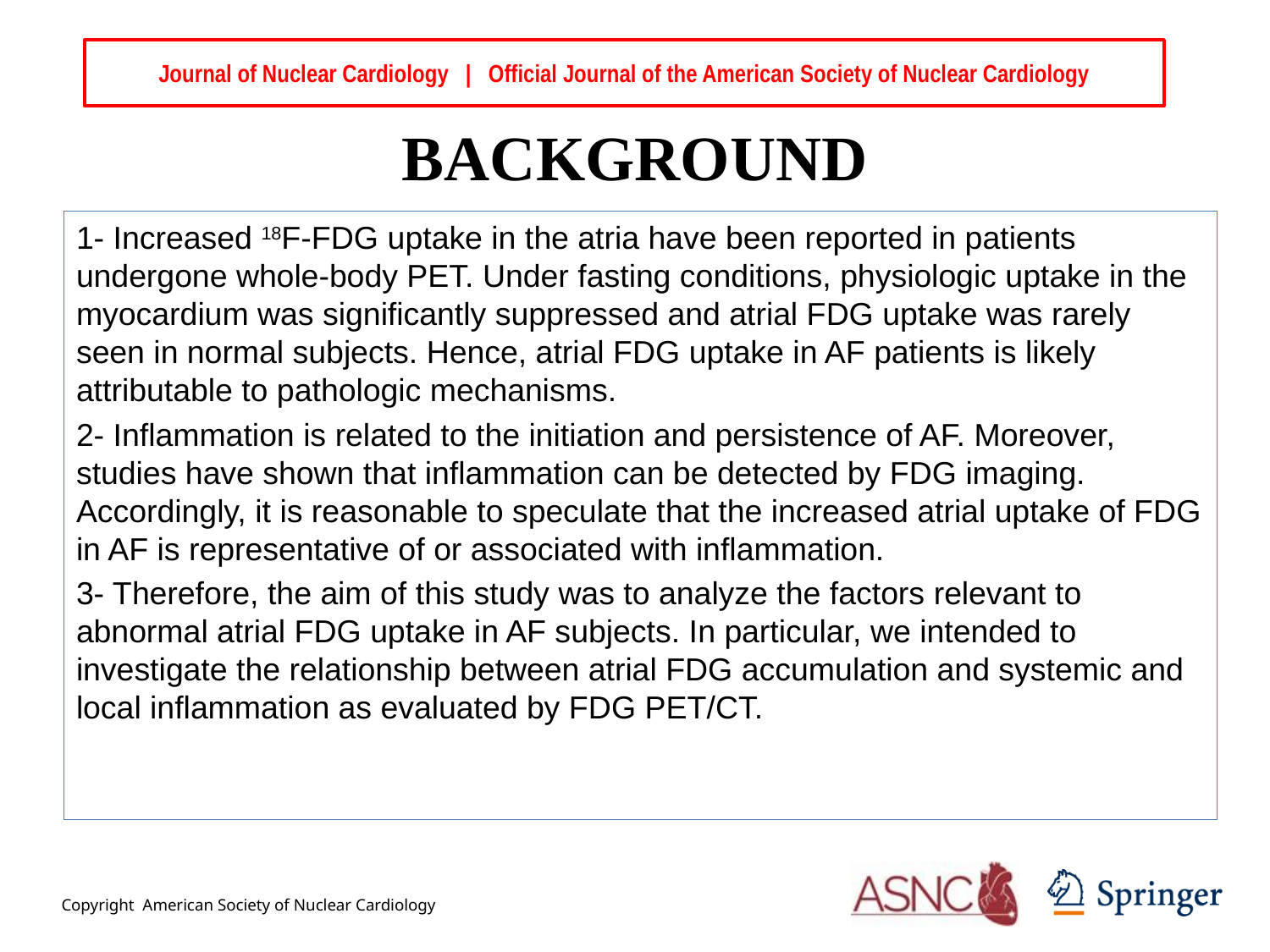

Journal of Nuclear Cardiology | Official Journal of the American Society of Nuclear Cardiology
# BACKGROUND
1- Increased 18F-FDG uptake in the atria have been reported in patients undergone whole-body PET. Under fasting conditions, physiologic uptake in the myocardium was significantly suppressed and atrial FDG uptake was rarely seen in normal subjects. Hence, atrial FDG uptake in AF patients is likely attributable to pathologic mechanisms.
2- Inflammation is related to the initiation and persistence of AF. Moreover, studies have shown that inflammation can be detected by FDG imaging. Accordingly, it is reasonable to speculate that the increased atrial uptake of FDG in AF is representative of or associated with inflammation.
3- Therefore, the aim of this study was to analyze the factors relevant to abnormal atrial FDG uptake in AF subjects. In particular, we intended to investigate the relationship between atrial FDG accumulation and systemic and local inflammation as evaluated by FDG PET/CT.
Copyright American Society of Nuclear Cardiology

## Slide 3
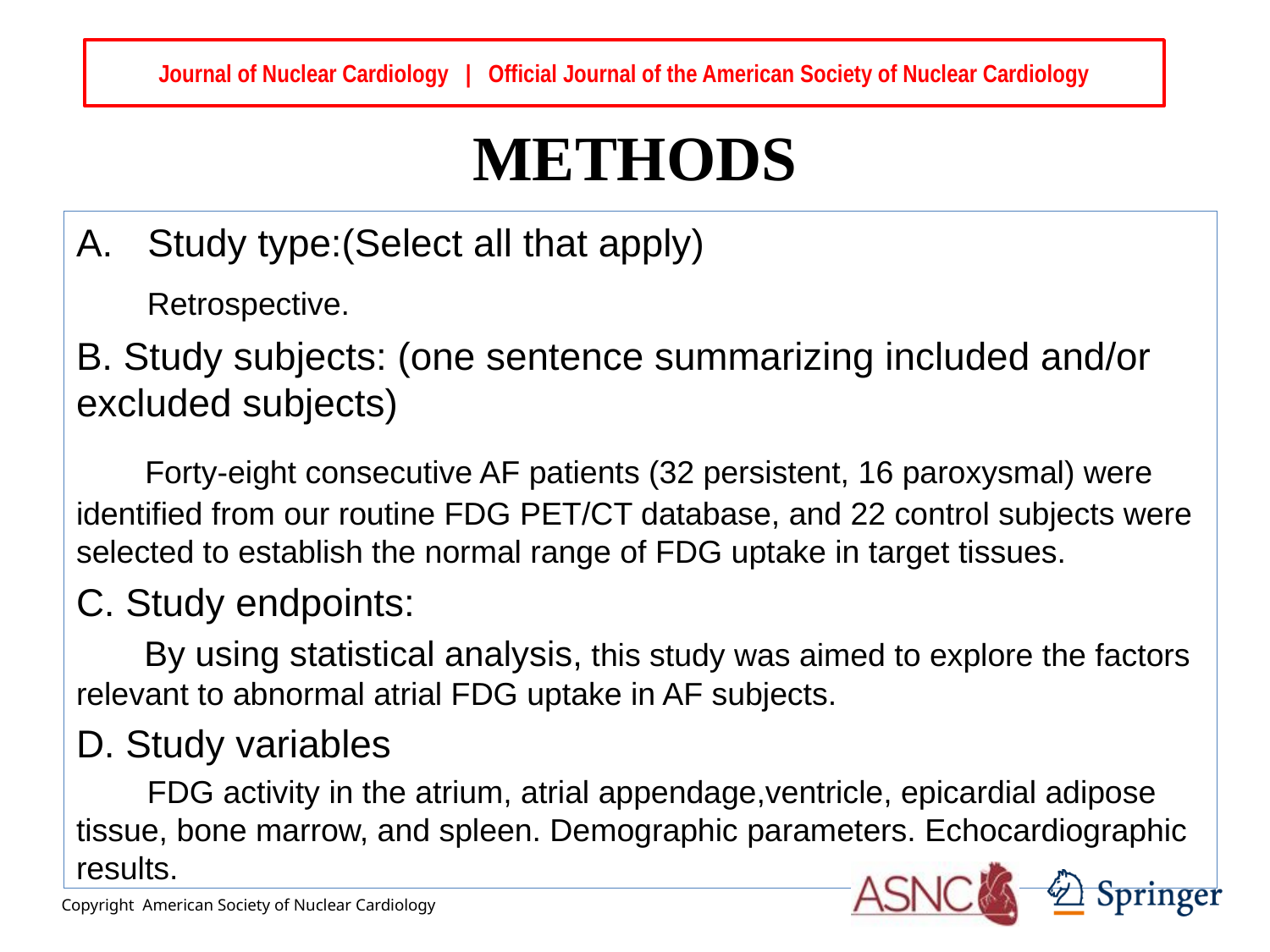

Journal of Nuclear Cardiology | Official Journal of the American Society of Nuclear Cardiology
# METHODS
Study type:(Select all that apply)
 Retrospective.
B. Study subjects: (one sentence summarizing included and/or excluded subjects)
 Forty-eight consecutive AF patients (32 persistent, 16 paroxysmal) were identified from our routine FDG PET/CT database, and 22 control subjects were selected to establish the normal range of FDG uptake in target tissues.
C. Study endpoints:
 By using statistical analysis, this study was aimed to explore the factors relevant to abnormal atrial FDG uptake in AF subjects.
D. Study variables
 FDG activity in the atrium, atrial appendage,ventricle, epicardial adipose tissue, bone marrow, and spleen. Demographic parameters. Echocardiographic results.
Copyright American Society of Nuclear Cardiology

## Slide 4
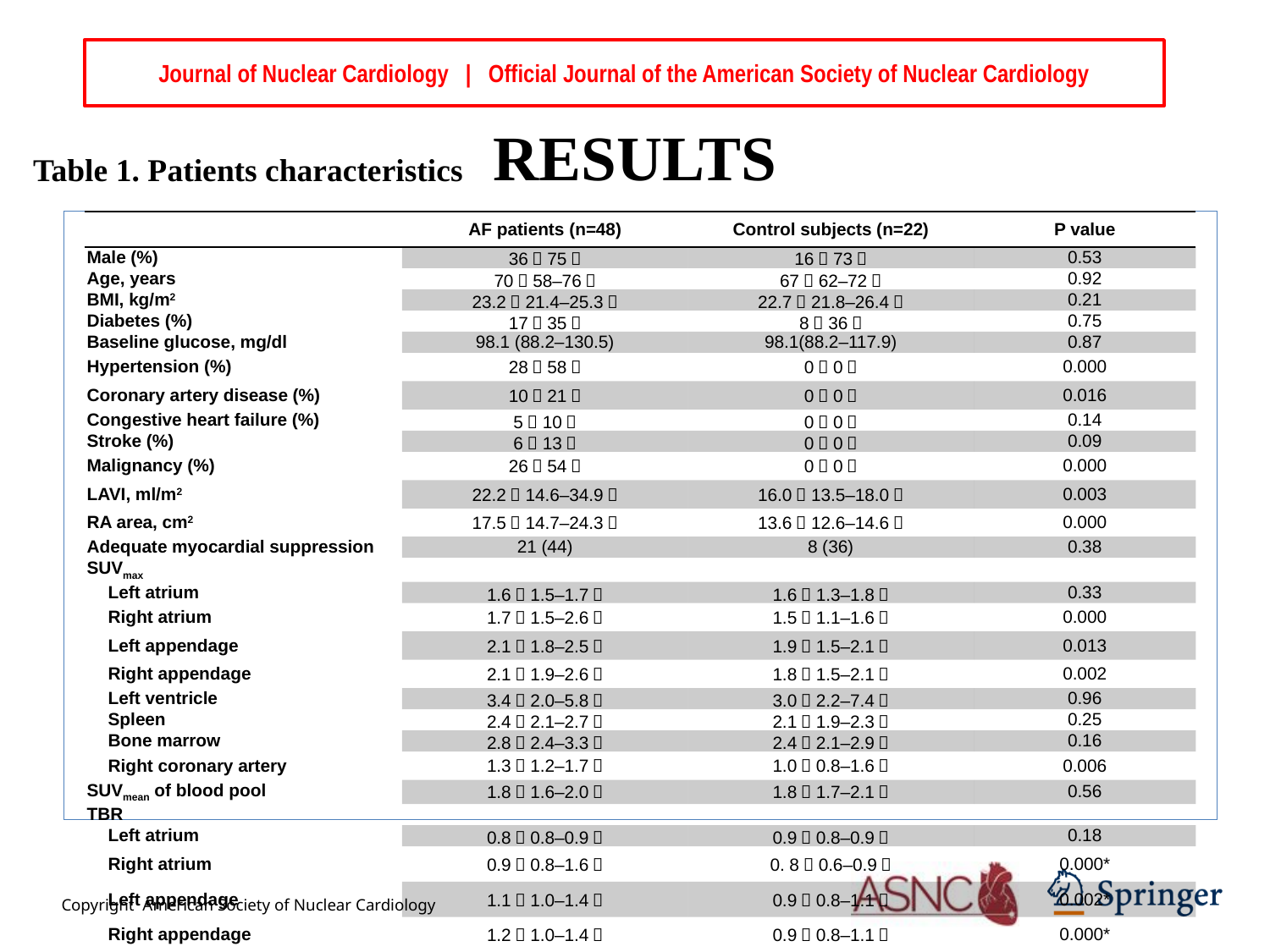

Journal of Nuclear Cardiology | Official Journal of the American Society of Nuclear Cardiology
# RESULTS
Table 1. Patients characteristics
| | AF patients (n=48) | Control subjects (n=22) | P value |
| --- | --- | --- | --- |
| Male (%) | 36（75） | 16（73） | 0.53 |
| Age, years | 70（58–76） | 67（62–72） | 0.92 |
| BMI, kg/m2 | 23.2（21.4–25.3） | 22.7（21.8–26.4） | 0.21 |
| Diabetes (%) | 17（35） | 8（36） | 0.75 |
| Baseline glucose, mg/dl | 98.1 (88.2–130.5) | 98.1(88.2–117.9) | 0.87 |
| Hypertension (%) | 28（58） | 0（0） | 0.000 |
| Coronary artery disease (%) | 10（21） | 0（0） | 0.016 |
| Congestive heart failure (%) | 5（10） | 0（0） | 0.14 |
| Stroke (%) | 6（13） | 0（0） | 0.09 |
| Malignancy (%) | 26（54） | 0（0） | 0.000 |
| LAVI, ml/m2 | 22.2（14.6–34.9） | 16.0（13.5–18.0） | 0.003 |
| RA area, cm2 | 17.5（14.7–24.3） | 13.6（12.6–14.6） | 0.000 |
| Adequate myocardial suppression | 21 (44) | 8 (36) | 0.38 |
| SUVmax | | | |
| Left atrium | 1.6（1.5–1.7） | 1.6（1.3–1.8） | 0.33 |
| Right atrium | 1.7（1.5–2.6） | 1.5（1.1–1.6） | 0.000 |
| Left appendage | 2.1（1.8–2.5） | 1.9（1.5–2.1） | 0.013 |
| Right appendage | 2.1（1.9–2.6） | 1.8（1.5–2.1） | 0.002 |
| Left ventricle | 3.4（2.0–5.8） | 3.0（2.2–7.4） | 0.96 |
| Spleen | 2.4（2.1–2.7） | 2.1（1.9–2.3） | 0.25 |
| Bone marrow | 2.8（2.4–3.3） | 2.4（2.1–2.9） | 0.16 |
| Right coronary artery | 1.3（1.2–1.7） | 1.0（0.8–1.6） | 0.006 |
| SUVmean of blood pool | 1.8（1.6–2.0） | 1.8（1.7–2.1） | 0.56 |
| TBR | | | |
| Left atrium | 0.8（0.8–0.9） | 0.9（0.8–0.9） | 0.18 |
| Right atrium | 0.9（0.8–1.6） | 0. 8（0.6–0.9） | 0.000\* |
| Left appendage | 1.1（1.0–1.4） | 0.9（0.8–1.1） | 0.002\* |
| Right appendage | 1.2（1.0–1.4） | 0.9（0.8–1.1） | 0.000\* |
Copyright American Society of Nuclear Cardiology

## Slide 5
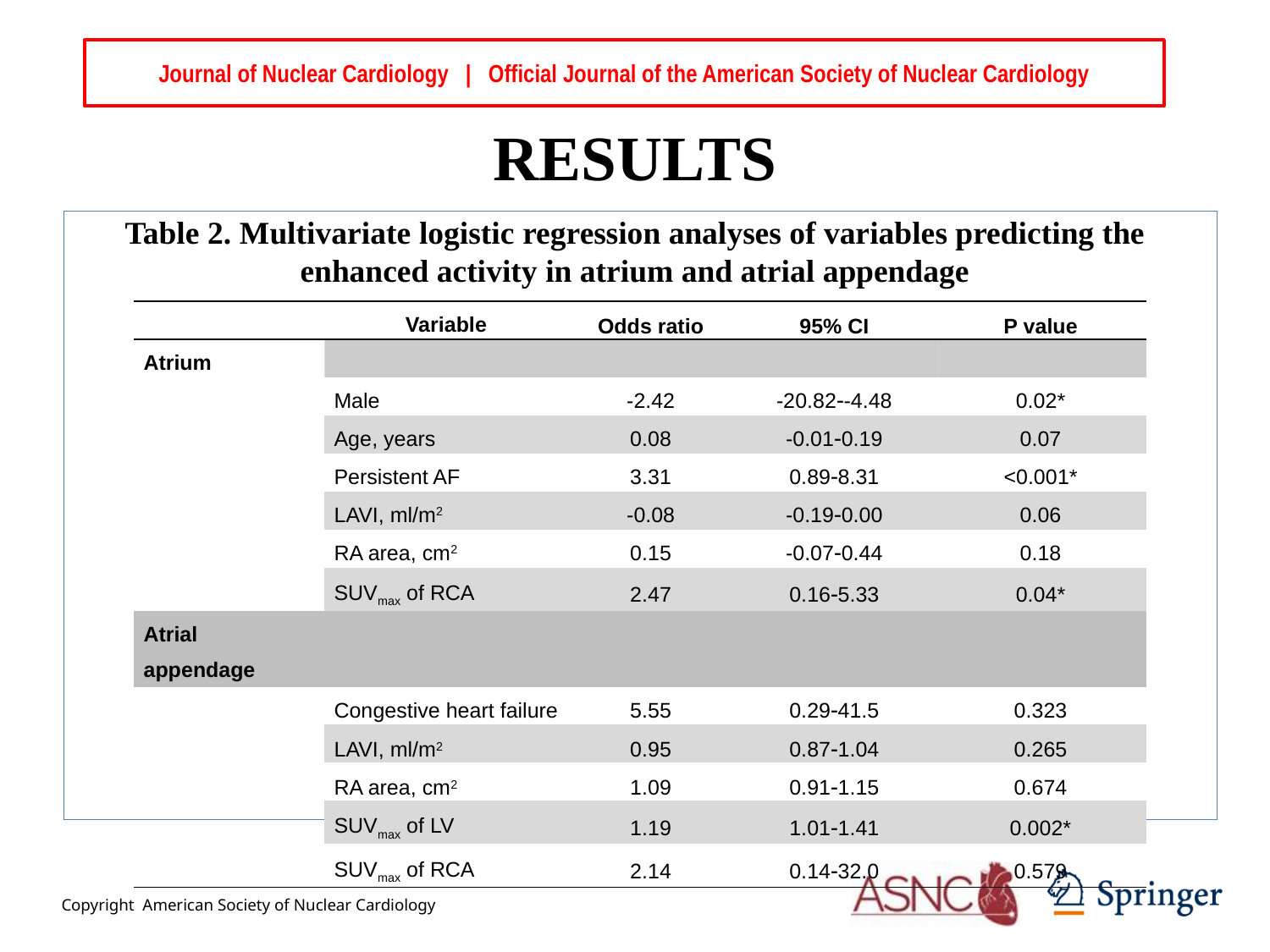

Journal of Nuclear Cardiology | Official Journal of the American Society of Nuclear Cardiology
# RESULTS
Table 2. Multivariate logistic regression analyses of variables predicting the enhanced activity in atrium and atrial appendage
| | Variable | Odds ratio | 95% CI | P value |
| --- | --- | --- | --- | --- |
| Atrium | | | | |
| | Male | -2.42 | -20.82−-4.48 | 0.02\* |
| | Age, years | 0.08 | -0.01−0.19 | 0.07 |
| | Persistent AF | 3.31 | 0.89−8.31 | <0.001\* |
| | LAVI, ml/m2 | -0.08 | -0.19−0.00 | 0.06 |
| | RA area, cm2 | 0.15 | -0.07−0.44 | 0.18 |
| | SUVmax of RCA | 2.47 | 0.16−5.33 | 0.04\* |
| Atrial appendage | | | | |
| | Congestive heart failure | 5.55 | 0.29−41.5 | 0.323 |
| | LAVI, ml/m2 | 0.95 | 0.87−1.04 | 0.265 |
| | RA area, cm2 | 1.09 | 0.91−1.15 | 0.674 |
| | SUVmax of LV | 1.19 | 1.01−1.41 | 0.002\* |
| | SUVmax of RCA | 2.14 | 0.14−32.0 | 0.579 |
Copyright American Society of Nuclear Cardiology

## Slide 6
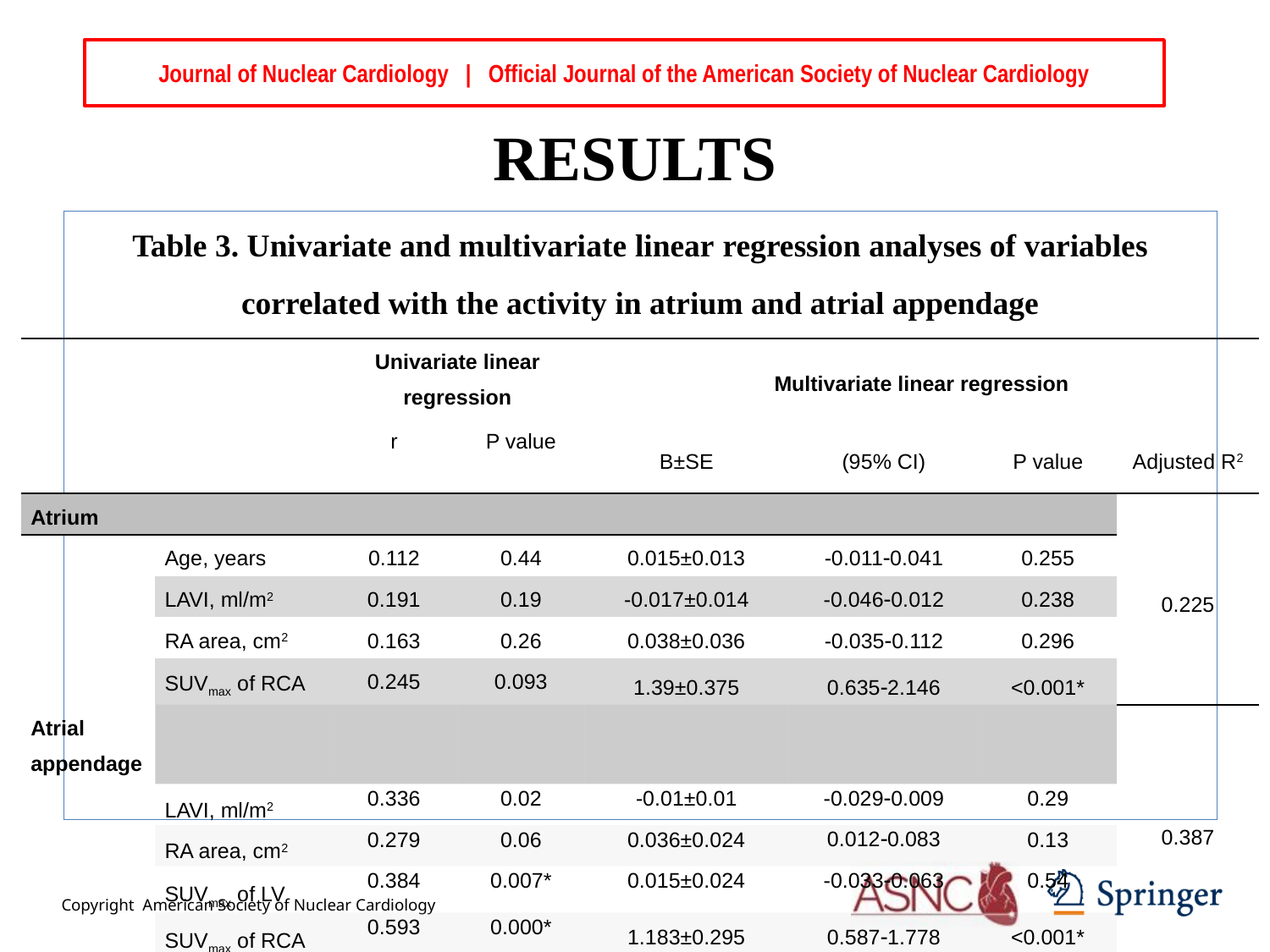

Journal of Nuclear Cardiology | Official Journal of the American Society of Nuclear Cardiology
# RESULTS
Table 3. Univariate and multivariate linear regression analyses of variables correlated with the activity in atrium and atrial appendage
| | | Univariate linear regression | | Multivariate linear regression | | | |
| --- | --- | --- | --- | --- | --- | --- | --- |
| | | r | P value | B±SE | (95% CI) | P value | Adjusted R2 |
| Atrium | | | | | | | 0.225 |
| | Age, years | 0.112 | 0.44 | 0.015±0.013 | -0.011−0.041 | 0.255 | |
| | LAVI, ml/m2 | 0.191 | 0.19 | -0.017±0.014 | -0.046−0.012 | 0.238 | |
| | RA area, cm2 | 0.163 | 0.26 | 0.038±0.036 | -0.035−0.112 | 0.296 | |
| | SUVmax of RCA | 0.245 | 0.093 | 1.39±0.375 | 0.635−2.146 | <0.001\* | |
| Atrial appendage | | | | | | | 0.387 |
| | LAVI, ml/m2 | 0.336 | 0.02 | -0.01±0.01 | -0.029−0.009 | 0.29 | |
| | RA area, cm2 | 0.279 | 0.06 | 0.036±0.024 | 0.012−0.083 | 0.13 | |
| | SUVmax of LV | 0.384 | 0.007\* | 0.015±0.024 | -0.033−0.063 | 0.54 | |
| | SUVmax of RCA | 0.593 | 0.000\* | 1.183±0.295 | 0.587−1.778 | <0.001\* | |
Copyright American Society of Nuclear Cardiology

## Slide 7
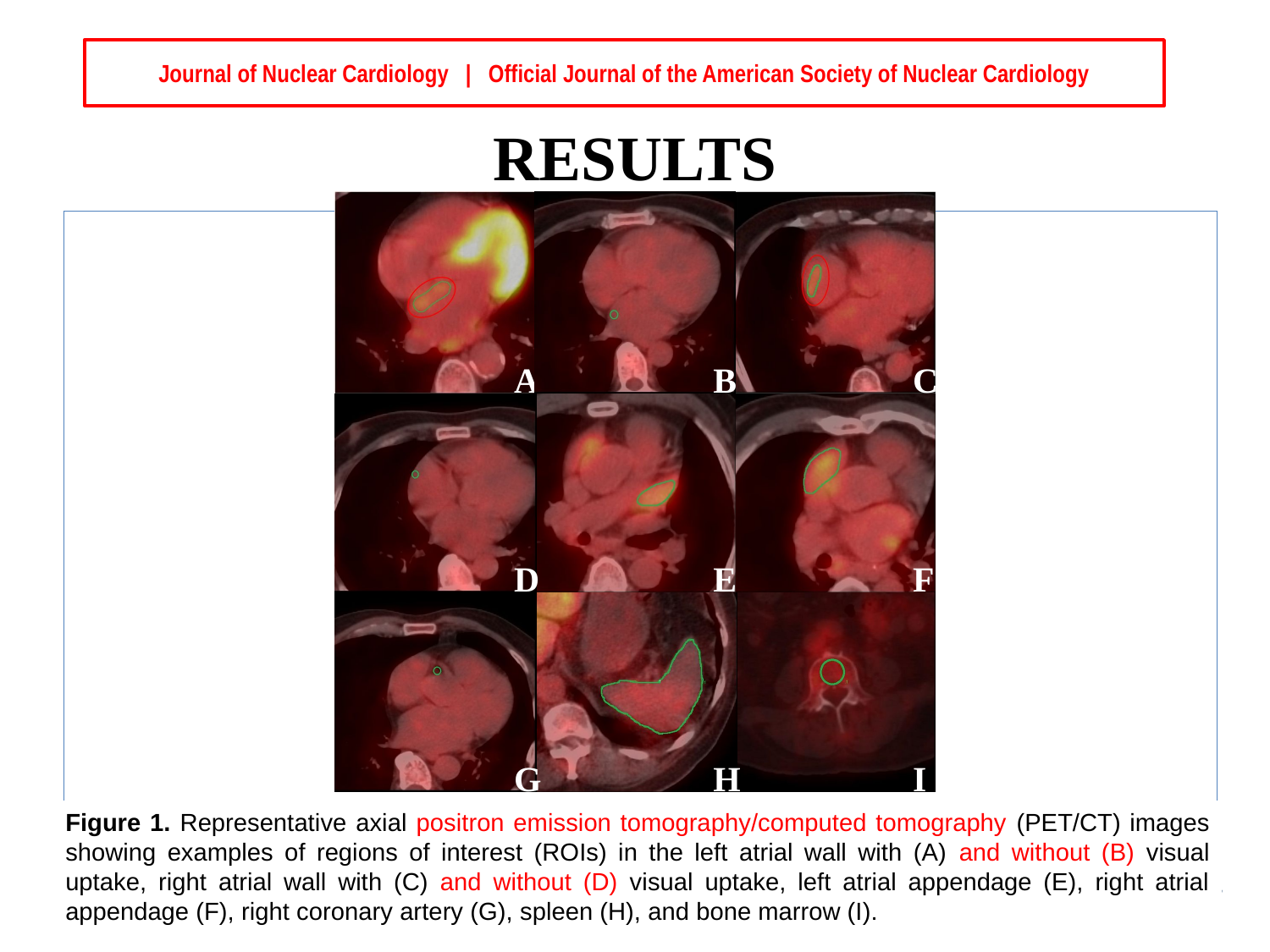

Journal of Nuclear Cardiology | Official Journal of the American Society of Nuclear Cardiology
# RESULTS
A
B
C
D
E
F
H
G
I
necessary
Figure 1. Representative axial positron emission tomography/computed tomography (PET/CT) images showing examples of regions of interest (ROIs) in the left atrial wall with (A) and without (B) visual uptake, right atrial wall with (C) and without (D) visual uptake, left atrial appendage (E), right atrial appendage (F), right coronary artery (G), spleen (H), and bone marrow (I).
Copyright American Society of Nuclear Cardiology

## Slide 8
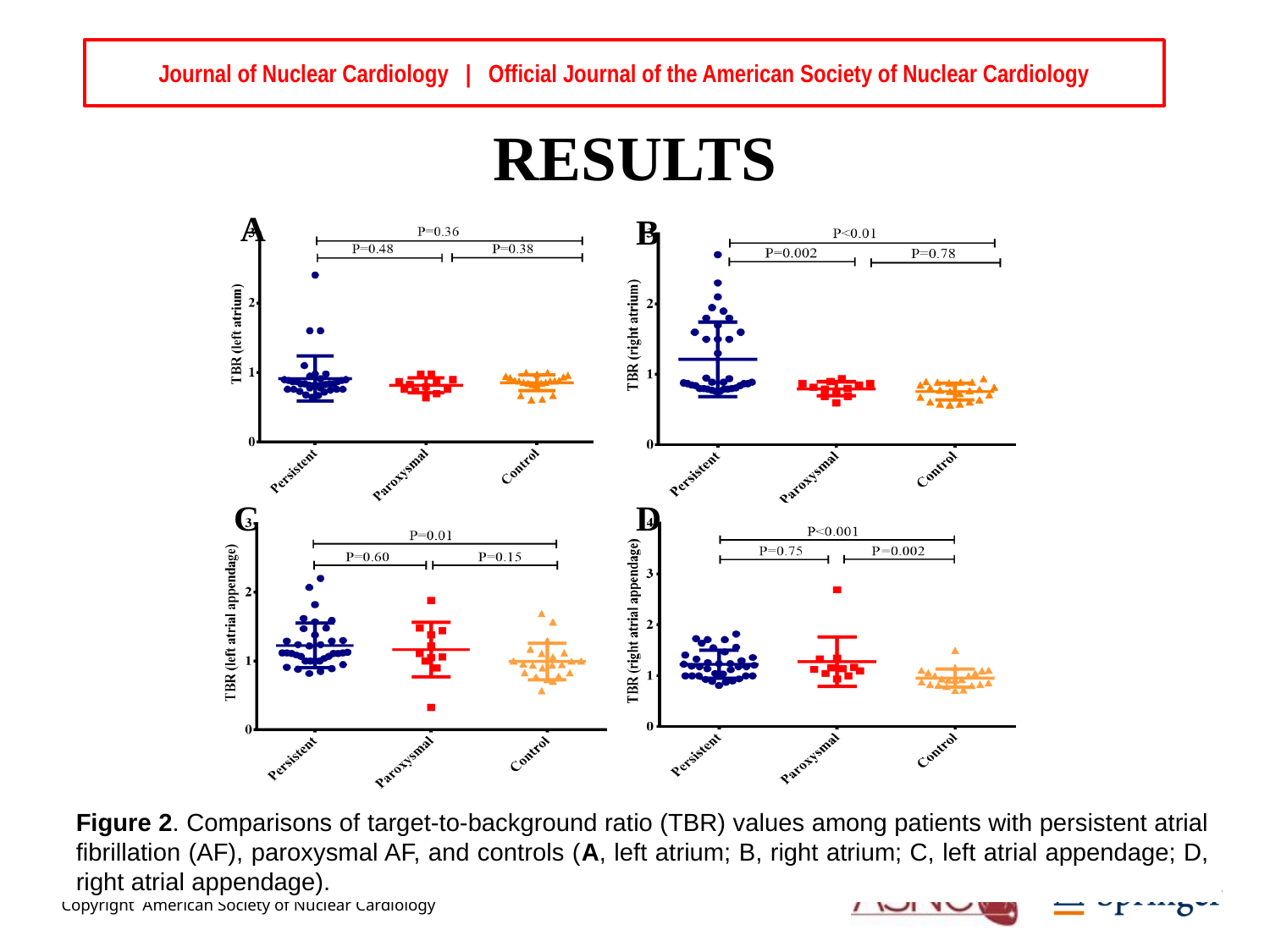

Journal of Nuclear Cardiology | Official Journal of the American Society of Nuclear Cardiology
# RESULTS
A
B
C
D
Figure 2. Comparisons of target-to-background ratio (TBR) values among patients with persistent atrial fibrillation (AF), paroxysmal AF, and controls (A, left atrium; B, right atrium; C, left atrial appendage; D, right atrial appendage).
Copyright American Society of Nuclear Cardiology

## Slide 9
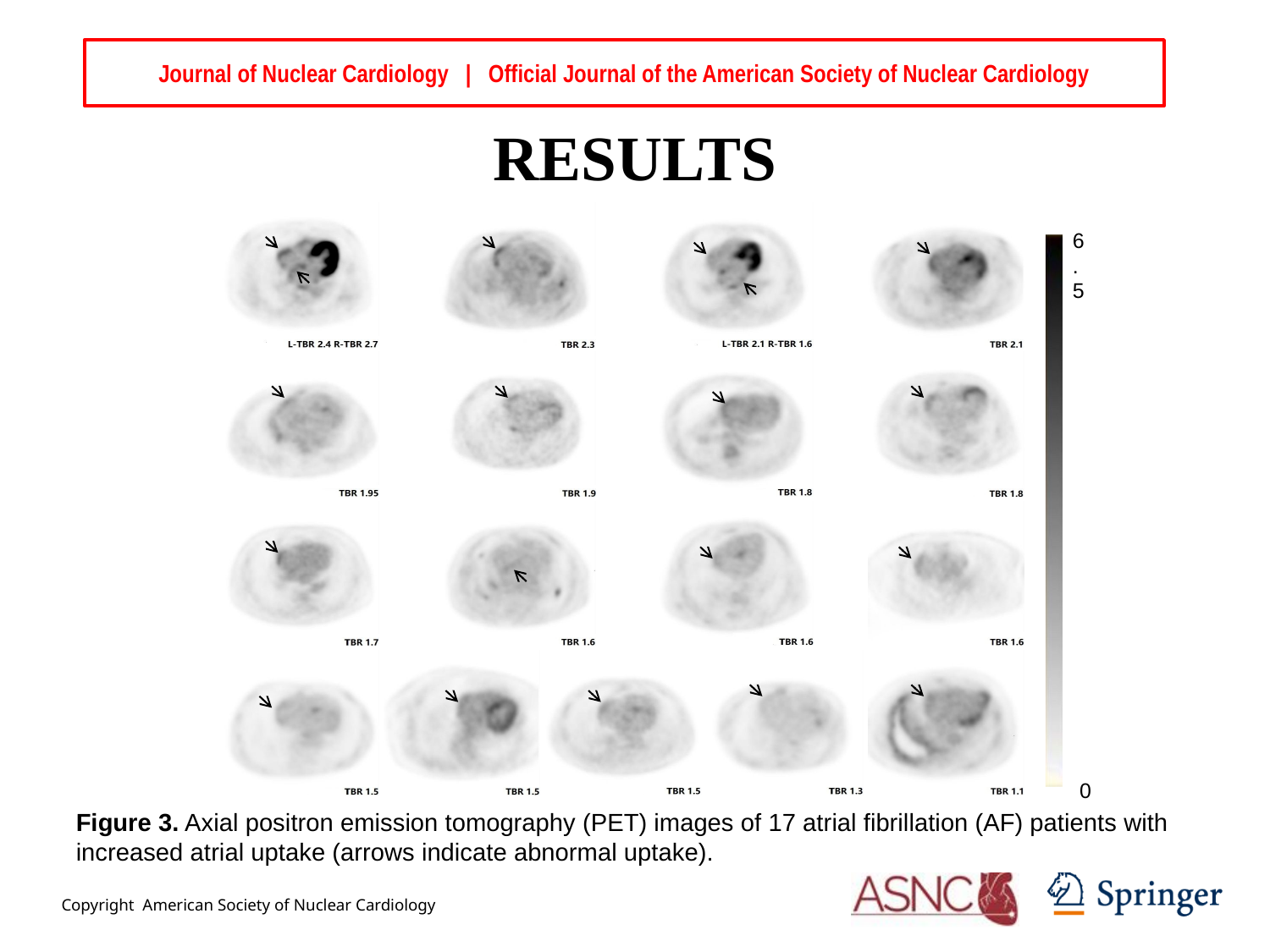

Journal of Nuclear Cardiology | Official Journal of the American Society of Nuclear Cardiology
# RESULTS
6.5
0
Figure 3. Axial positron emission tomography (PET) images of 17 atrial fibrillation (AF) patients with increased atrial uptake (arrows indicate abnormal uptake).
Copyright American Society of Nuclear Cardiology

## Slide 10
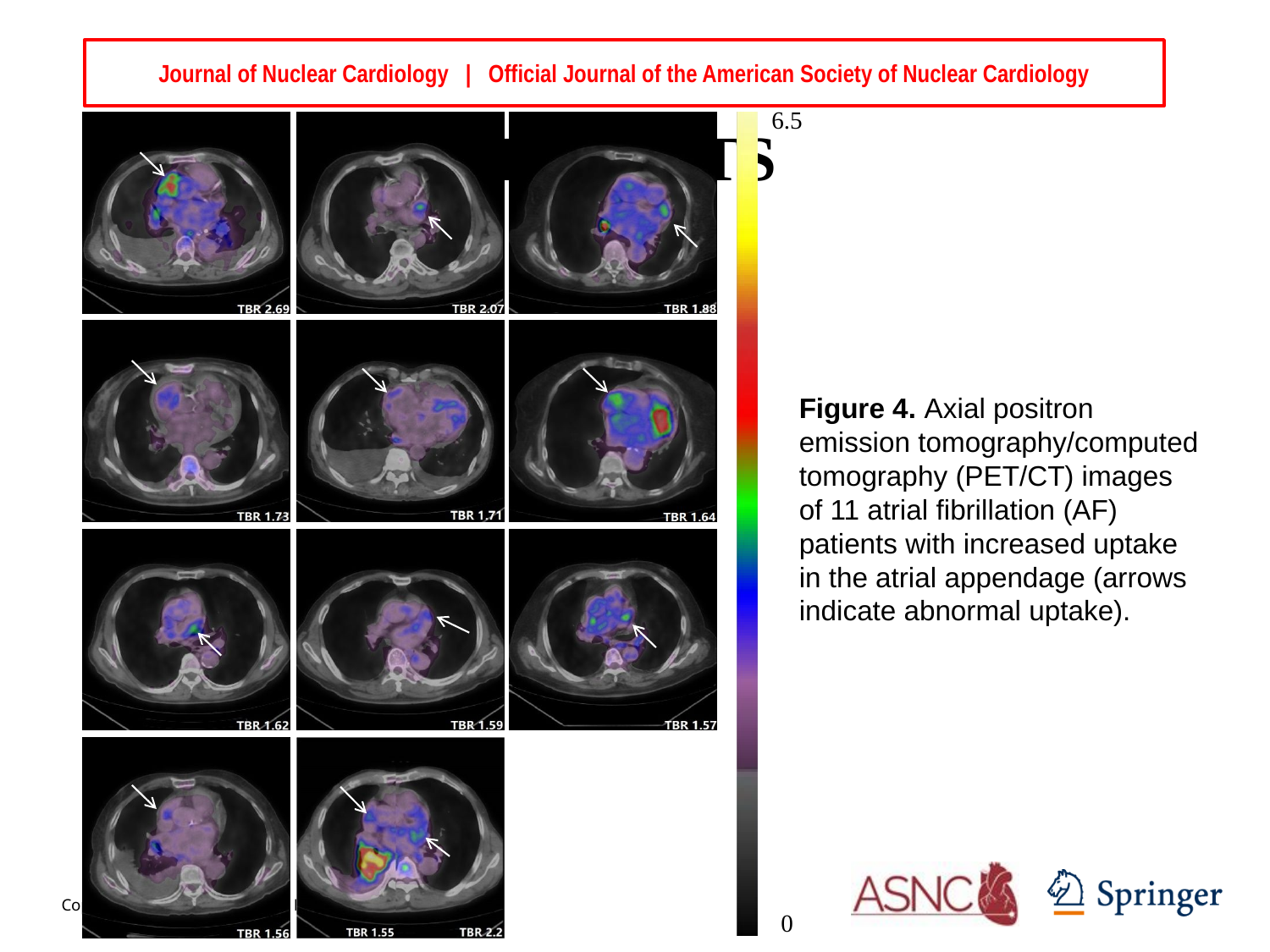

Journal of Nuclear Cardiology | Official Journal of the American Society of Nuclear Cardiology
6.5
0
# RESULTS
Figure 4. Axial positron emission tomography/computed tomography (PET/CT) images of 11 atrial fibrillation (AF) patients with increased uptake in the atrial appendage (arrows indicate abnormal uptake).
Copyright American Society of Nuclear Cardiology

## Slide 11
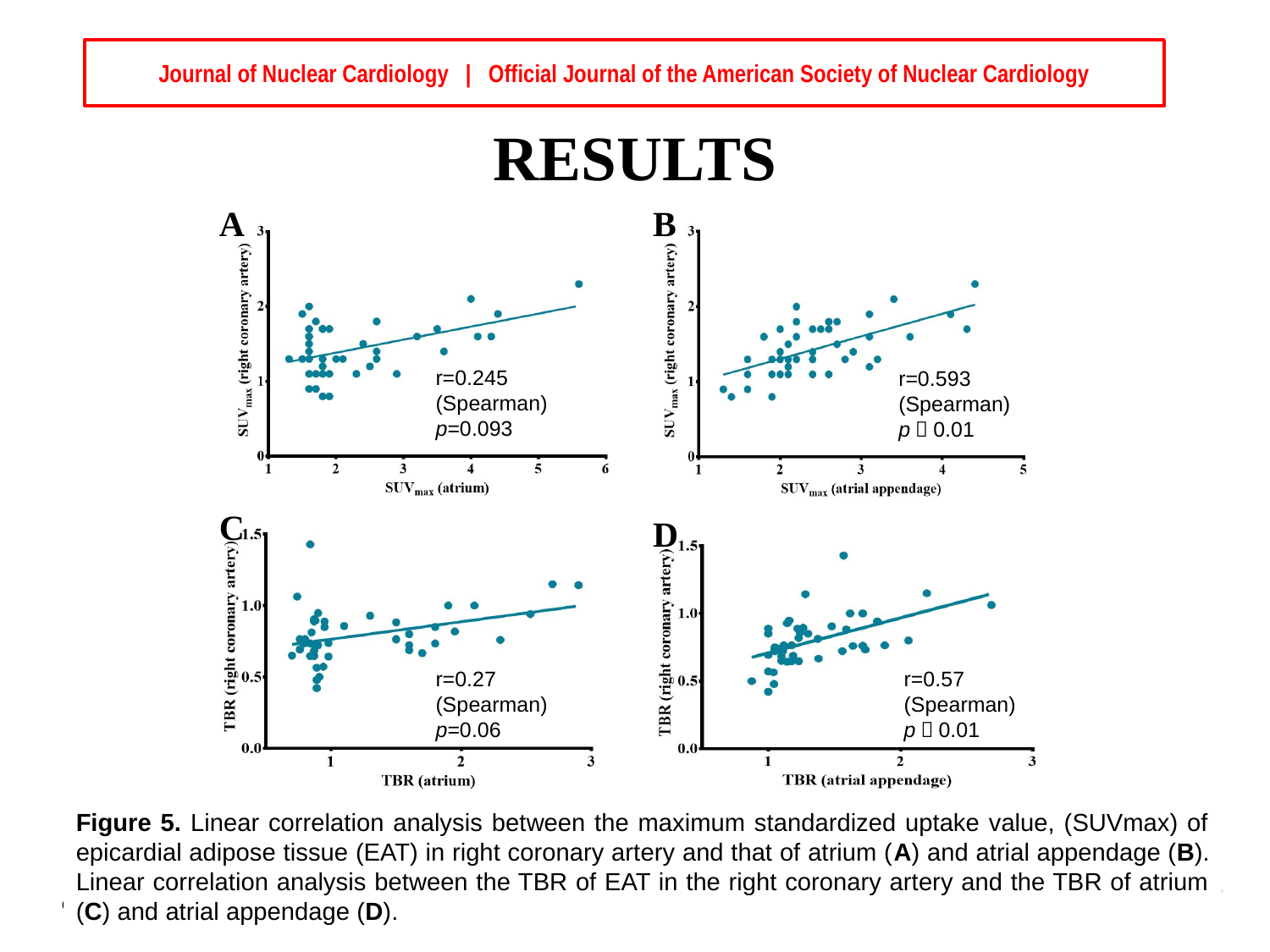

Journal of Nuclear Cardiology | Official Journal of the American Society of Nuclear Cardiology
# RESULTS
A
B
r=0.245 (Spearman)
p=0.093
r=0.593 (Spearman)
p＜0.01
C
r=0.27 (Spearman)
p=0.06
D
r=0.57 (Spearman)
p＜0.01
Figure 5. Linear correlation analysis between the maximum standardized uptake value, (SUVmax) of epicardial adipose tissue (EAT) in right coronary artery and that of atrium (A) and atrial appendage (B). Linear correlation analysis between the TBR of EAT in the right coronary artery and the TBR of atrium (C) and atrial appendage (D).
Copyright American Society of Nuclear Cardiology

## Slide 12
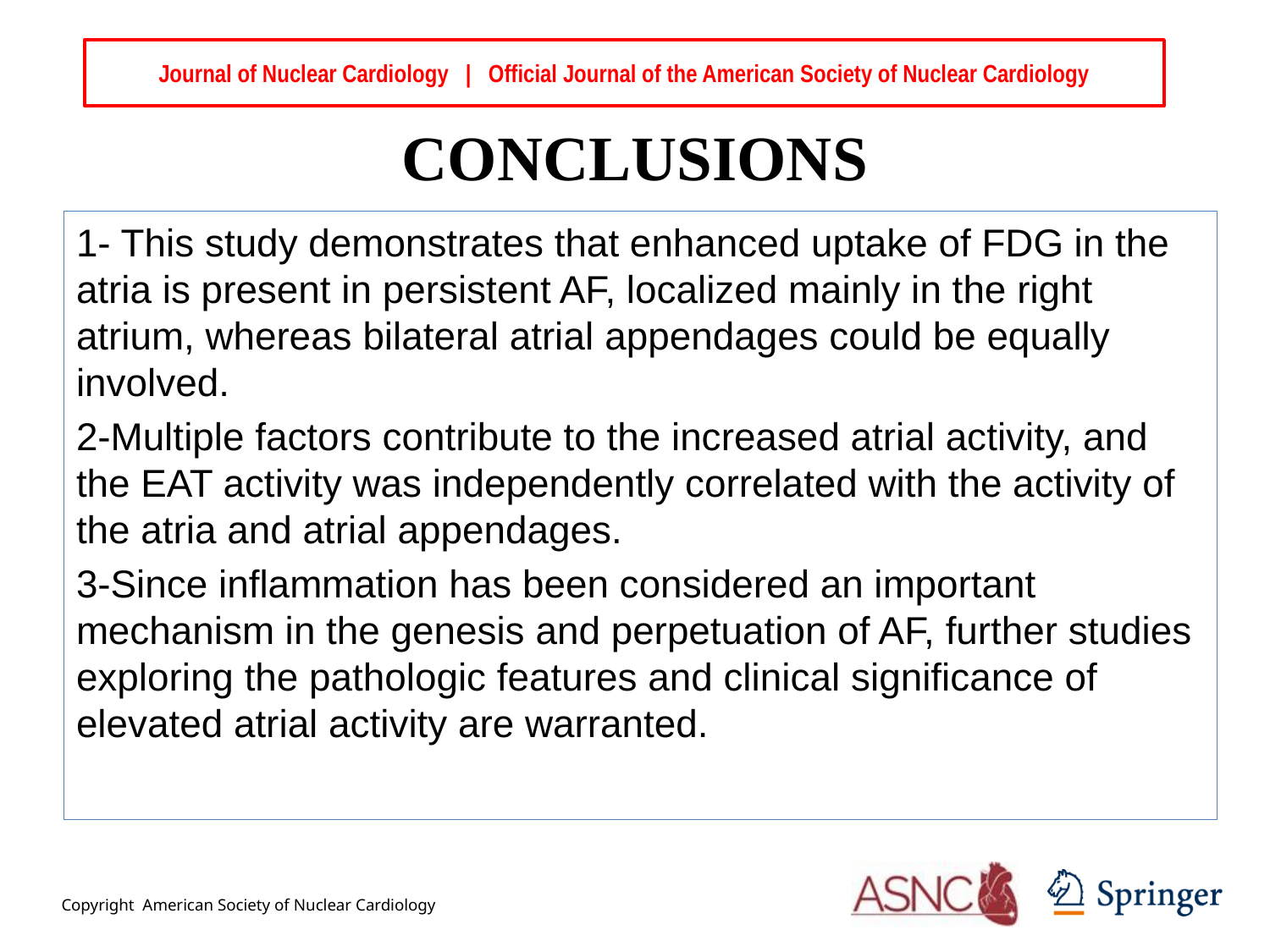

Journal of Nuclear Cardiology | Official Journal of the American Society of Nuclear Cardiology
# CONCLUSIONS
1- This study demonstrates that enhanced uptake of FDG in the atria is present in persistent AF, localized mainly in the right atrium, whereas bilateral atrial appendages could be equally involved.
2-Multiple factors contribute to the increased atrial activity, and the EAT activity was independently correlated with the activity of the atria and atrial appendages.
3-Since inflammation has been considered an important mechanism in the genesis and perpetuation of AF, further studies exploring the pathologic features and clinical significance of elevated atrial activity are warranted.
Copyright American Society of Nuclear Cardiology
